# Cannabidiol Differentially Modulates Salinomycin Cytotoxicity in Human Cell Lines

**DOI:** 10.3390/molecules31214039

**Published:** 2026-11-09

**Authors:** Oliwia Kończak, Tatiana Wojciechowicz, Ovinuchi Ejiohuo, Paula Żakowicz, Oskar Łętowski, Agnieszka Ścibior, Joanna Zeyland, Lidia Radko

**Affiliations:** 1 Department of Biochemistry and Biotechnology, Poznan University of Life Sciences, Dojazd Street 11, 60-632 Poznan, Poland; oliwia.konczak@up.poznan.pl (O.K.); ovinuchi.ejiohuo@up.poznan.pl (O.E.); 2 Department of Animal Physiology, Biochemistry and Biostructure, Faculty of Veterinary Medicine and Animal Sciences, Poznan University of Life Sciences, 60-637 Poznan, Poland; tatiana.wojciechowicz@up.poznan.pl; 3 Students Scientific Society of Veterinary Medicine, Section of Veterinary Pharmacology and Toxicology “Paracelsus”, Faculty of Veterinary Medicine and Animal Sciences, Poznan University of Life Sciences, 60-637 Poznan, Poland; paulazakowicz@gmail.com (P.Ż.); oskar.letowsky@gmail.com (O.Ł.); 4 Department of Biomedicine and Environmental Research, The John Paul II Catholic University of Lublin, Konstantynów 1J, 20-708 Lublin, Poland; agnieszka.scibior@kul.pl; 5 Department of Preclinical Sciences and Infectious Diseases, Faculty of Veterinary Medicine and Animal Sciences, Poznan University of Life Sciences, 60-637 Poznan, Poland; 6 Experimental Center, National Veterinary Research Institute, 24-100 Pulawy, Poland

**Keywords:** salinomycin, cannabidiol, interaction, in vitro, human cells

## Abstract

Salinomycin, an ionophore coccidiostat widely used in livestock production, has attracted growing interest as a potential anticancer agent, although its clinical application remains limited by pronounced organ-specific toxicity. Cannabidiol (CBD), a non-psychoactive cannabinoid with documented cytoprotective properties, is increasingly consumed, making concurrent exposure plausible, yet its interaction remains unexplored. This study evaluated the cytotoxicity of salinomycin (0.39–50 μg/mL), alone and combined with two non-cytotoxic CBD concentrations (1.56 and 3.125 μg/mL), in three human cell lines—embryonic kidney-derived HEK-293, hepatoblastoma-derived HepG2, and neuroblastoma-derived SH-SY5Y—using assays of mitochondrial and lysosomal activity, membrane integrity, total protein content, and DNA synthesis, complemented by ROS measurement, apoptosis/necrosis evaluation, morphology, and an exploratory combination index analysis. CBD modulated salinomycin cytotoxicity in a strictly cell-line-dependent manner. In HEK-293 cells, it attenuated cytotoxicity, as reflected by uniformly higher IC_50_ values, reduced ROS production, and lower median fluorescence intensities of Hoechst 33342 and propidium iodide, whereas in the tumor-derived HepG2 and SH-SY5Y lines, it enhanced cytotoxicity irrespective of the CBD concentration applied, accompanied by increased ROS generation and more pronounced cell death. As all three lines are immortalized or tumor-derived, these observations describe cell-line-specific responses and cannot be extrapolated to normal renal, hepatic, or neuronal tissue.

## 1. Introduction

Livestock farming is inherently associated with the use of veterinary medicinal products. The occurrence of diseases on farms, including coccidiosis caused by parasitic protozoa of the genus *Eimeria* spp., necessitates the use of veterinary drugs known as coccidiostats, including those belonging to the group of polyether ionophore antibiotics, such as salinomycin. Salinomycin is a polyether monocarboxylic acid produced by the fermentation of the bacteria *Streptomyces albus* and *Streptomyces aureofaciens*. Its mechanism of action is related to its high affinity for monovalent cations (Na^+^, K^+^), which disrupts their transmembrane transport. Owing to its ability to form lipophilic complexes with these ions, salinomycin transports them across biological membranes independently of ion channels, thereby disturbing the physiological concentration gradients between the intracellular and extracellular compartments, as well as between the cytoplasm and the mitochondrial matrix. The drug shows high antimicrobial activity against Gram-positive bacteria, *Clostridium perfringens*, mycobacteria, and some filamentous fungi [1].

In recent years, salinomycin has attracted considerable interest as a potential anticancer agent [2], but its species- and organ-specific toxicity remains the principal obstacle to clinical development [3]. Clinical poisoning has been documented in turkeys, pre-ruminant calves, adult cattle, horses, cats and dogs, with pigs comparatively less sensitive, and toxicity has been reported in some non-target species at feed concentrations below the maximum permitted for chickens for fattening—evidence of pronounced interspecies variability and of a narrow safety margin [4]. Reported clinical signs include cardiovascular disturbances such as elevated blood pressure and myocardial degeneration, together with anorexia, weakness, ataxia, and paralysis [1,4,5].

In poisonings of farm animals, extensive acute renal tubular necrosis has been described [6], and pigmented nephropathy secondary to myoglobinuria has been documented [7], while the first documented case of human salinomycin poisoning confirmed its capacity to cause multi-organ damage [8]. Mechanistically, the nephrotoxicity of this compound has been attributed both to a direct ionophoric effect on renal tubular cells and to secondary kidney damage resulting from skeletal muscle rhabdomyolysis [6,7].

Neurotoxicity is the most extensively documented sign of salinomycin intoxication and is widely recognized as a factor limiting its therapeutic development [9]. Van der Linde-Sipman et al. described an outbreak in cats fed contaminated feed, in which the animals developed polyneuropathy confirmed by morphological examination [10], and Aleman et al. reported poisoning in six horses with extensive vacuolation of dorsal root ganglion neurons and of nerve-root axons, followed in survivors by chronic muscle atrophy, weakness, and ataxia [11]. Neurological symptoms were also prominent in the single reported case of human poisoning [8].

Hepatotoxicity is reported less frequently than cardio-, myo-, and neurotoxicity. Salinomycin was detected in the livers of poisoned cats, confirming distribution to this organ [10], and liver damage accompanied the predominant cardiac and neurological disturbances in the human case, although the authors emphasized how limited the human toxicological data remain [8]. In swine, where salinomycin toxicity is potentiated by the CYP450 inhibitor tiamulin, the liver does not appear to be the primary target organ, pointing to interspecies differences in the tissue distribution of salinomycin toxicity [7].

It should be noted that errors made by breeders—such as the prophylactic use of high doses of salinomycin throughout the entire production cycle, prolonged and ineffective therapy, and failure to observe withdrawal periods—contribute to the presence of this drug in food products of animal origin (eggs, meat, tissues), thereby posing a risk to consumer health by toxicological effects, such as teratogenicity, hepatotoxicity, or neurotoxicity. Additionally, such practices among breeders contribute to the development of drug resistance and the occurrence of toxic interactions with concurrently administered medications [12].

The evaluation of pharmacologically active compounds with respect to their safety for animals, consumers, and the environment falls under the responsibility of the European Food Safety Authority (EFSA). This safety assessment process also encompasses the establishment of maximum residue limits (MRLs) for coccidiostats in food products derived from both target and non-target animal species [5]. Maximum residue limits for salinomycin in relevant food products of animal origin are established under Commission Implementing Regulation (EU) 2017/1914 concerning the authorization of sodium salinomycin as a feed additive for chickens for fattening and chickens reared for laying, and repealing Regulations (EC) No 1852/2003 and (EC) No 1463/2004 [13].

A central approach to safeguarding public health relies on employing natural bioactive substances to mitigate the detrimental effects of antibiotic residues found in food and environmental matrices. The rising popularity and expanding market presence of hemp-derived cannabidiol (CBD) oil products may help address this challenge. Derived naturally from *Cannabis sativa*, CBD lacks psychoactive properties and addictive potential [14,15]. It should be stressed, however, that the absence of intoxicating effects is not in itself evidence of toxicological safety, and CBD has been reported to exert concentration-dependent cytotoxic effects in some cell models. Research findings have further revealed its wide-ranging cytoprotective effects in experimental models involving toxic renal injury [16,17,18,19,20,21], liver [20,22,23], and nervous system [24,25].

In the present study, three human cell lines were used as in vitro models of renal (HEK-293), hepatic (HepG2), and neural (SH-SY5Y) tissue. All three are immortalized or tumor-derived, and none reproduces the physiology of the corresponding normal tissue. HEK-293 was derived from human embryonic kidney cells transformed with DNA from human adenovirus type 5 [26]. It is among the most widely used models in studies of renal cytotoxicity and pharmacology, although its suitability as a surrogate for differentiated renal epithelium has been questioned [27]. HepG2, derived from a human hepatoma, retains many hepatocyte functions and is one of the most widely used systems for assessing the hepatic effects of chemicals and drugs [28,29]. SH-SY5Y, a human neuroblastoma cell line, is widely used in neurobiological research [30] and in the screening of potentially neurotoxic agents and candidate drugs for central nervous system disorders [31]. Used in this way, these lines are suitable for defining effective concentration ranges and for probing mechanisms of action. They do not support conclusions about normal renal, hepatic, or neuronal tissue.

The aim of this study is to examine the effect of cannabidiol on salinomycin-induced cytotoxicity in these three cell lines. Over a 72 h exposure, cells were treated with salinomycin alone (0.39–50 μg/mL; 0.50–64.7 μM) or in combination with CBD at 1.56 μg/mL (SAL + C1) or 3.125 μg/mL (SAL + C2). Mitochondrial function, lysosomal integrity, plasma membrane damage, total protein content, and DNA synthesis were measured, and IC_50_ values were determined, with analyses of reactive oxygen species generation, cell death, and morphology. Interactions were quantified using the combination index.

## 2. Results

### 2.1. Cytotoxicity of Cannabidiol Alone

**Figure 1:**
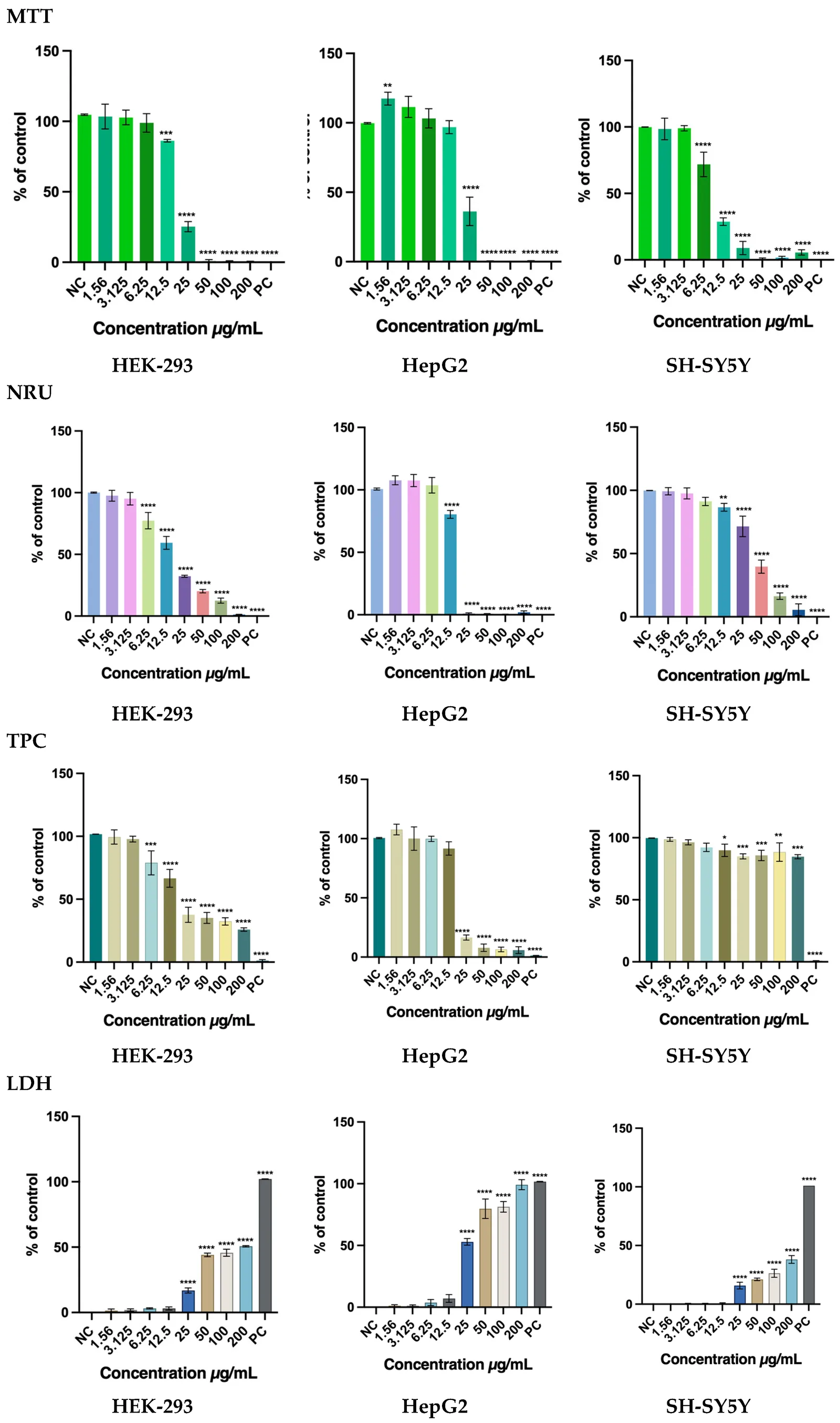

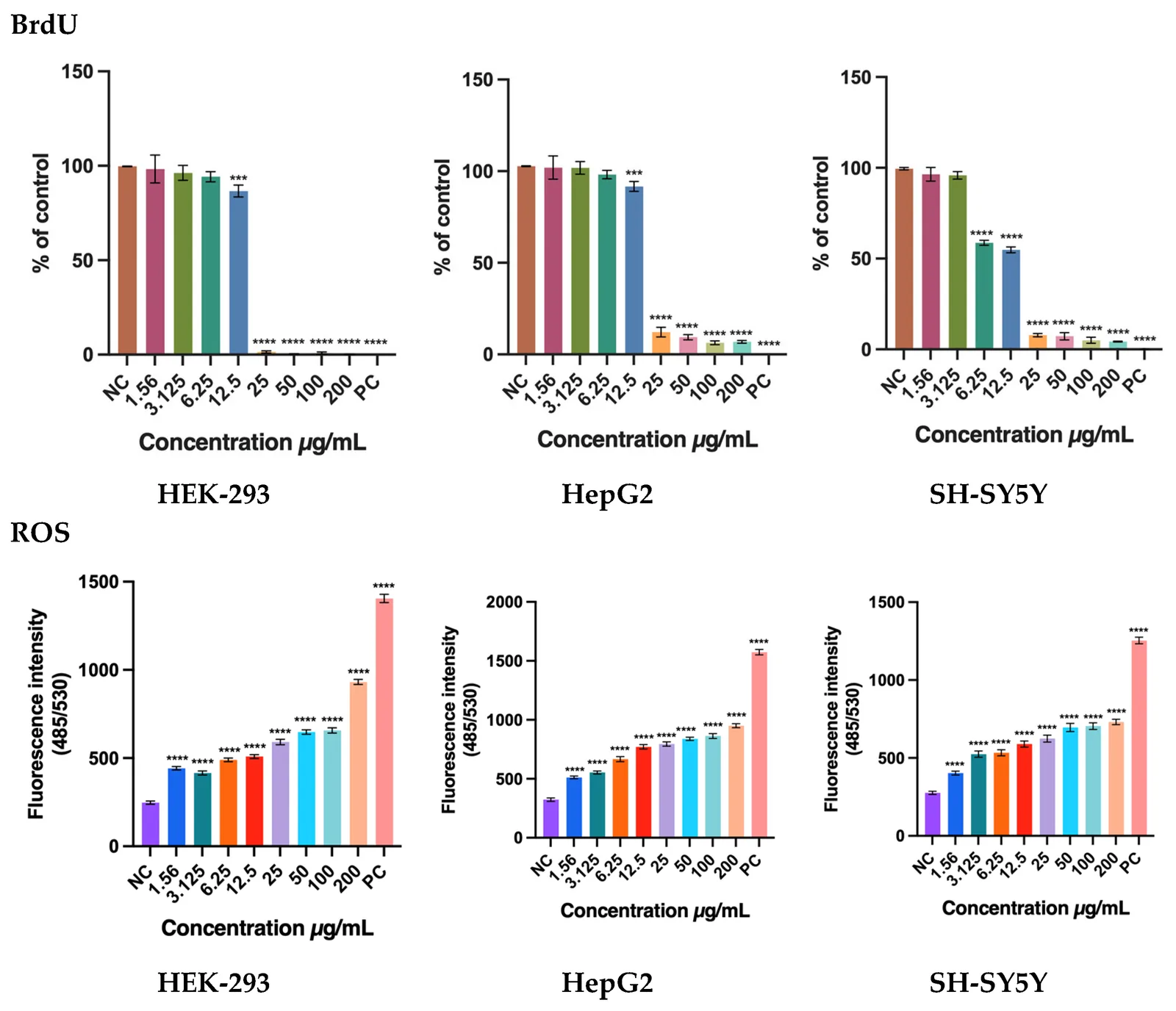
Effect of cannabidiol (CBD) alone at concentrations of 1.56–200 μg/mL (4.96–636 μM) on HEK-293, HepG2, and SH-SY5Y cells after 72 h of exposure, assessed using five cytotoxicity assays—mitochondrial activity (MTT), lysosomal activity (NRU), total protein content (TPC), cell membrane integrity (LDH), and DNA synthesis (BrdU) together with reactive oxygen species production (ROS). Results of the MTT, NRU, TPC, and BrdU assays are expressed as a percentage of the negative control (0.1% DMSO in medium), and those of the LDH assay as a percentage of maximal release defined by the Triton X-100 positive control; ROS data are expressed as fluorescence intensity (485/530 nm) and are not normalized to cell number or viability. Data are presented as mean ± SD, *n* = 3 independent experiments. NC, negative control; PC, positive control (1% Triton X-100 for the cytotoxicity assays, 500 μM H2O2 for ROS). Significance relative to the negative control was assessed using one-way ANOVA with Dunnett’s post hoc test: * *p* ≤ 0.05, ** *p* ≤ 0.01, *** *p* ≤ 0.001, **** *p* ≤ 0.0001.

Cannabidiol alone was evaluated over the range 1.56–200 μg/mL (4.96–636 μM) in HEK-293, HepG2, and SH-SY5Y cells using the MTT, NRU, TPC, LDH, and BrdU assays, together with measurement of ROS production (Figure 1). At the two concentrations subsequently used in combination with salinomycin, 1.56 and 3.125 μg/mL (4.96 and 9.94 μM), cannabidiol did not significantly inhibit mitochondrial activity, lysosomal activity, total protein content, or DNA synthesis in any of the three cell lines, and LDH release did not exceed 2% of maximal release. The only significant deviation from the negative control at these two concentrations was observed in HepG2 cells, where 1.56 μg/mL increased the MTT signal to 118% of control (*p* ≤ 0.01); no corresponding increase was significant in any other assay or cell line.

Significant inhibition first appeared at 6.25 μg/mL or higher in all cases. In HEK-293 cells, the earliest significant reductions were recorded at 6.25 μg/mL in the NRU and TPC assays and at 12.5 μg/mL in the MTT and BrdU assays. In HepG2 cells at 12.5 μg/mL in the NRU and BrdU assays and at 25 μg/mL in the MTT and TPC assays, and in SH-SY5Y cells at 6.25 μg/mL in the MTT and BrdU assays and at 12.5 μg/mL in the NRU and TPC assays. LDH release first increased significantly at 25 μg/mL in all three cell lines. In SH-SY5Y cells, the total protein content did not fall below 84% of control at any concentration tested, so cannabidiol produced less than 20% inhibition of this endpoint across the whole range.

Reactive oxygen species production followed a different pattern from the viability endpoints. Cannabidiol significantly increased ROS production across all concentrations tested in all three cell lines (*p* ≤ 0.0001). At the two concentrations subsequently used in combination with salinomycin, the increase was 1.79- and 1.68-fold of the negative control in HEK-293 cells, 1.58- and 1.71-fold in HepG2 cells, and 1.46- and 1.90-fold in SH-SY5Y cells. ROS production increased further with concentration, reaching 3.77-, 2.94-, and 2.65-fold of the negative control at 200 μg/mL (Figure 1). The two concentrations used in combination therefore increased reactive oxygen species production without inhibiting any viability endpoint.

Median-effect analysis of the cannabidiol-alone data (Table 1) gave Dm values of 11.6–144.5 μg/mL (36.9–459.5 μM) in HEK-293 cells, 25.0–32.8 μg/mL (79.5–104.3 μM) in HepG2 cells, and 12.3–203.2 μg/mL (39.1–646.2 μM) in SH-SY5Y cells, counting only the endpoints that yielded a valid median-effect relationship. The lowest of these, 11.6 μg/mL for DNA synthesis in HEK-293 cells, is 3.7-fold above the highest cannabidiol concentration used in combination with salinomycin. Concentrations at which the measured signal equaled or exceeded the negative control yield fa ≤ 0 and cannot be placed on a median-effect plot; such points were excluded from the regressions, which accounts for the smaller number of concentrations entering the HepG2 MTT, NRU, and TPC fits (*n* = 5, 5, and 7, respectively; Table 1). Median-effect plots were linear for twelve of the fifteen assay–cell line combinations (r = 0.90–1.00). The remaining three did not yield a valid median-effect relationship; the combination index values derived for them are reported but flagged.

### 2.2. The Effects of CBD on Salinomycin’s Activity in the HEK-293 Cell Line

**Figure 2:**
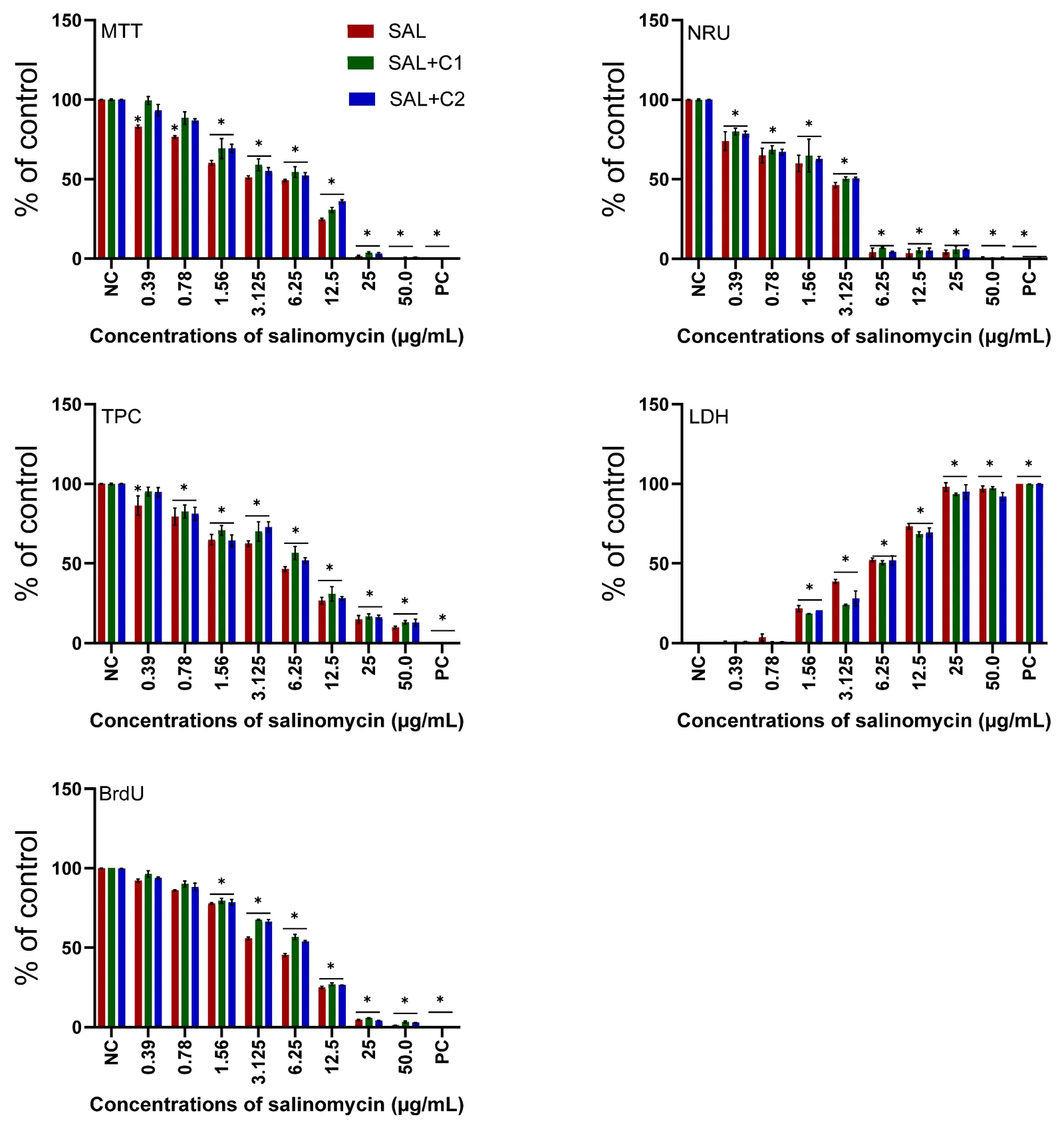
Effects of salinomycin (SAL) alone in the range of 0.39–50 μg/mL (0.50–64.7 μM) and in combination with cannabidiol (CBD) at concentrations of 1.56 μg/mL (4.96 μM; SAL + C1) and 3.125 μg/mL (9.94 μM; SAL + C2) on HEK-293 cell lines after 72 h of exposure, assessed using five assays: mitochondrial activity (MTT assay), lysosomal activity (NRU assay), proliferation (TPC assay), cell membrane integrity (LDH assay), and DNA synthesis (BrdU assay). Results are expressed as a percentage of the negative control (NC = 100%) and presented as mean ± SD (standard deviation), *n* = 3 (the number of independent experiments, each performed in six technical replicates per concentration), NC—negative control; PC—positive control. Statistical significance was assessed by comparing each treatment group (SAL, SAL + C1, SAL + C2) with NC. The horizontal line above each concentration group spans the treatment that differed significantly from NC, and the asterisk above the line indicates *p* ≤ 0.05 for that comparison.

Salinomycin inhibited mitochondrial activity, lysosomal activity, and cell proliferation in HEK-293 cells starting at a concentration of 0.39 μg/mL (*p* ≤ 0.05). At 1.56 μg/mL, the drug inhibited DNA synthesis and induced cell membrane disruption (Figure 2). The presence of CBD in both the SAL + C1 and SAL + C2 mixtures increased the drug concentration required to inhibit the evaluated endpoints. In renal cells exposed to these mixtures, inhibition of mitochondrial activity and cell proliferation was only observed at higher drug concentrations—1.56 μg/mL and 0.78 μg/mL, respectively (Figure 2).

In HEK-293 cells, the lowest IC_50_ values for the drug and its two mixtures with CBD were observed in the NRU assay, compared to the MTT, TPC, LDH, and BrdU assays. In the MTT, TPC, and BrdU assays, the IC_50_ values of both mixtures were 2.3- to 2.6-fold higher than those of salinomycin alone, but with *n* = 3 this increase reached statistical significance only in the BrdU assay; in the NRU and LDH assays no differences were found between salinomycin alone and either mixture (Table 2). The increase in the MTT and TPC assays was of the same direction and magnitude as in the BrdU assay and fell just short of the significance threshold (Tukey *p* = 0.067–0.19), so the attenuation of salinomycin cytotoxicity in this cell line rests on the consistent displacement of the complete concentration–response curves (Figure 2) rather than on the IC_50_ point estimates alone. The full ANOVA and Tukey output for every comparison in Table 2 is given in Table S2. Although lysosomal activity (NRU assay) in HEK-293 cells was already significantly reduced at the lowest tested concentration (0.39 μg/mL; approximately 75–80% of control, *p* ≤ 0.05), this initial decrease was comparatively modest. The NRU dose–response curve was markedly steeper than that of the other endpoints, with values dropping below 10% of control between 3.125 and 6.25 μg/mL; this steep decline, rather than the magnitude of the initial effect, accounts for the lowest IC_50_ values recorded for the NRU assay in HEK-293 cells (Table 2).

**Figure 3:**
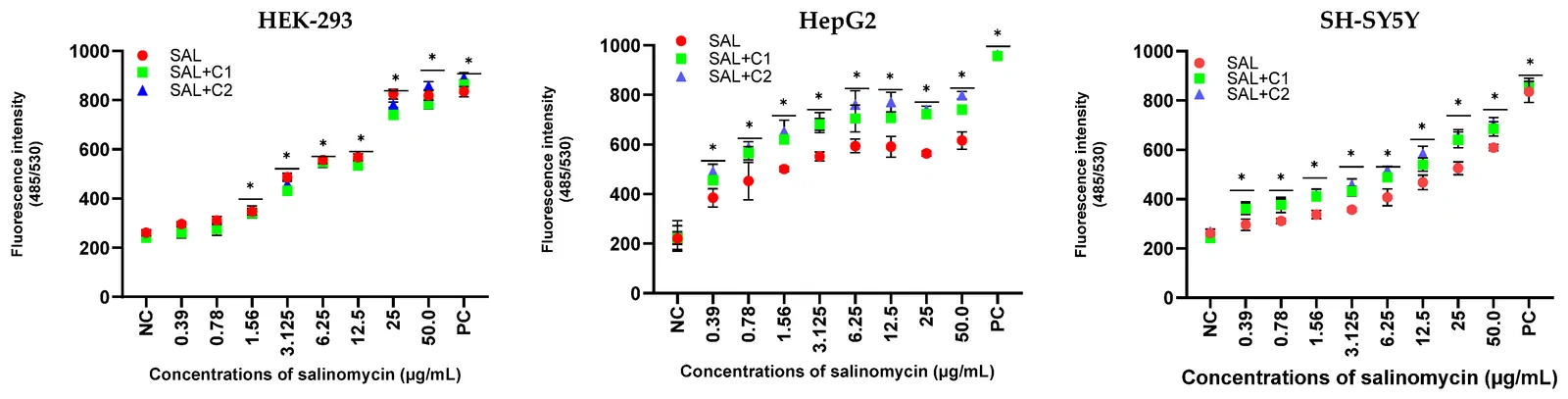
Production of reactive oxygen species (ROS) after 72 h of exposure of HEK-293, HepG2, and SH-SY5Y cells to salinomycin (SAL) alone in the range of 0.39–50 μg/mL (0.50–64.7 μM) and in combination with cannabidiol (CBD) at concentrations of 1.56 μg/mL (4.96 μM; SAL + C1) and 3.125 μg/mL (9.94 μM; SAL + C2). NC (negative control); PC (positive control). Data are presented as mean ± SD (standard deviation), *n* = 3 (refers to independent experiments, each performed in six technical replicates). Fluorescence values are expressed as a percentage of the vehicle-matched negative control and were not normalized to cell number. Statistical significance was assessed comparing each treatment group with NC; the horizontal line above each concentration group spans the treatment that differed significantly from NC, and the asterisk indicates * *p* ≤ 0.05 for that comparison.

An increase in reactive oxygen species (ROS) production was observed in HEK-293 cultures starting at a salinomycin concentration of 1.56 μg/mL. The presence of CBD in both mixtures resulted in lower ROS production than salinomycin alone at the same concentration (Figure 3).

**Figure 4:**
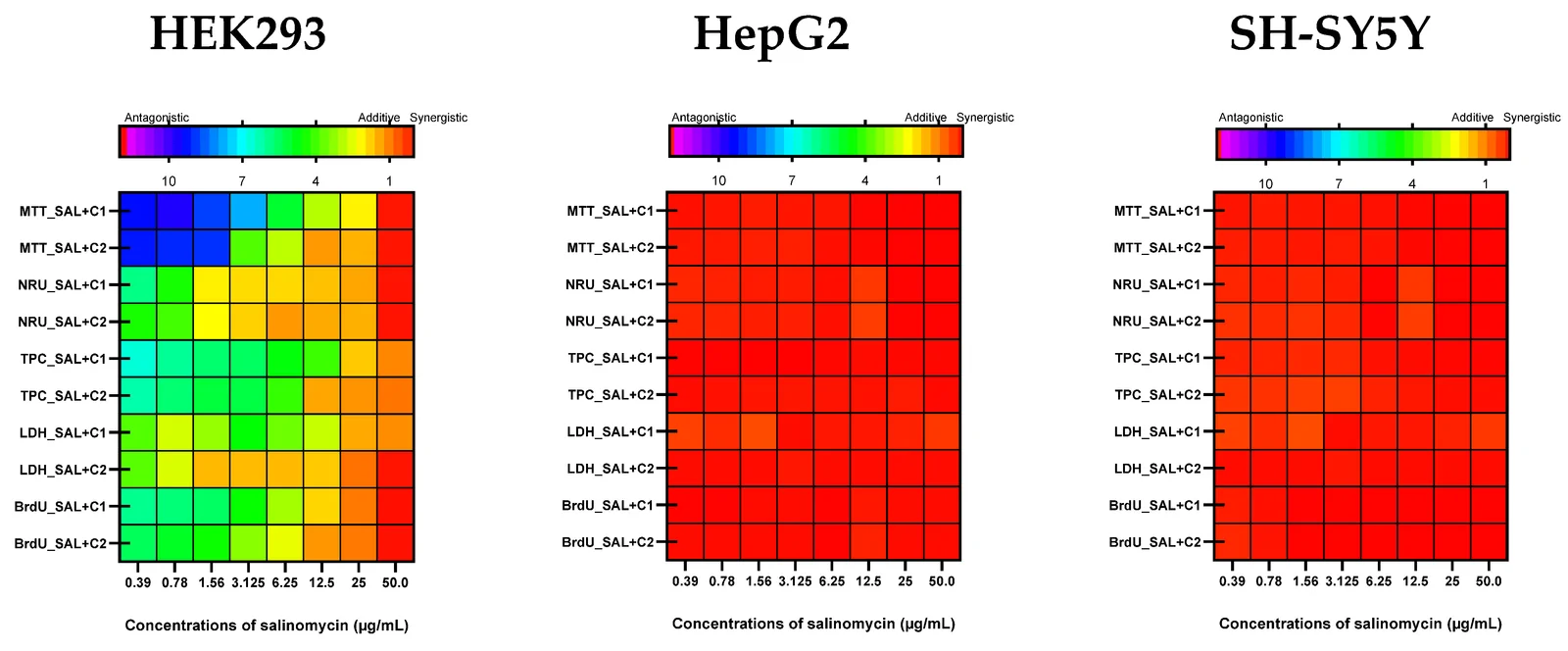
Heat maps illustrate the effects of the combination of SAL at concentrations of 0.39–50 μg/mL (0.50–64.7 μM) with CBD at a low concentration (1.56 μg/mL, 4.96 μM; SAL + C1) and a higher concentration (3.125 μg/mL, 9.94 μM; SAL + C2) in HEK-293, HepG2, and SH-SY5Y cell cultures across five different assays after 72 h of exposure. In the combination index (CI) heat map, red indicates synergy (CI < 0.9), green indicates additive effects (0.9 ≤ CI < 1.1), and blue indicates antagonism (CI ≥ 1.1). CI values were calculated from median-effect plots constructed for salinomycin alone, for CBD alone and for the SAL + CBD mixtures, each normalized to its own vehicle-matched negative control (NC, DMSO-containing medium without SAL or CBD = 100%); no positive control (PC) was used in this analysis. The numerical CI values corresponding to the colour scale are provided in Supplementary Table S1. The CI analysis was performed as an exploratory, non-constant-ratio combination analysis; its assumptions and limitations are described in Section 4.11.

The nature of the interactions between SAL and CBD depended on the concentration of both substances in the mixtures. Antagonism predominated: CI values exceeded 1.1 for both mixtures in all five assays at salinomycin concentrations from 0.39 to 12.5 μg/mL, and at 25 μg/mL in every case except the LDH assay with SAL + C2, where the interaction was additive (CI 1.07). At the highest salinomycin concentration (50 μg/mL) the interaction reversed to synergy in the MTT, NRU and BrdU assays for both mixtures (CI 0.13–0.20) and in the LDH assay for SAL + C2 (CI 0.18), whereas it remained antagonistic in the TPC and LDH assays with SAL + C1 (CI 1.24 and 1.31) and was additive in the TPC assay with SAL + C2 (CI 1.09). The corresponding heat maps are shown in Figure 4, and numerical CI values for every condition are given in Table S1.

**Figure 5:**
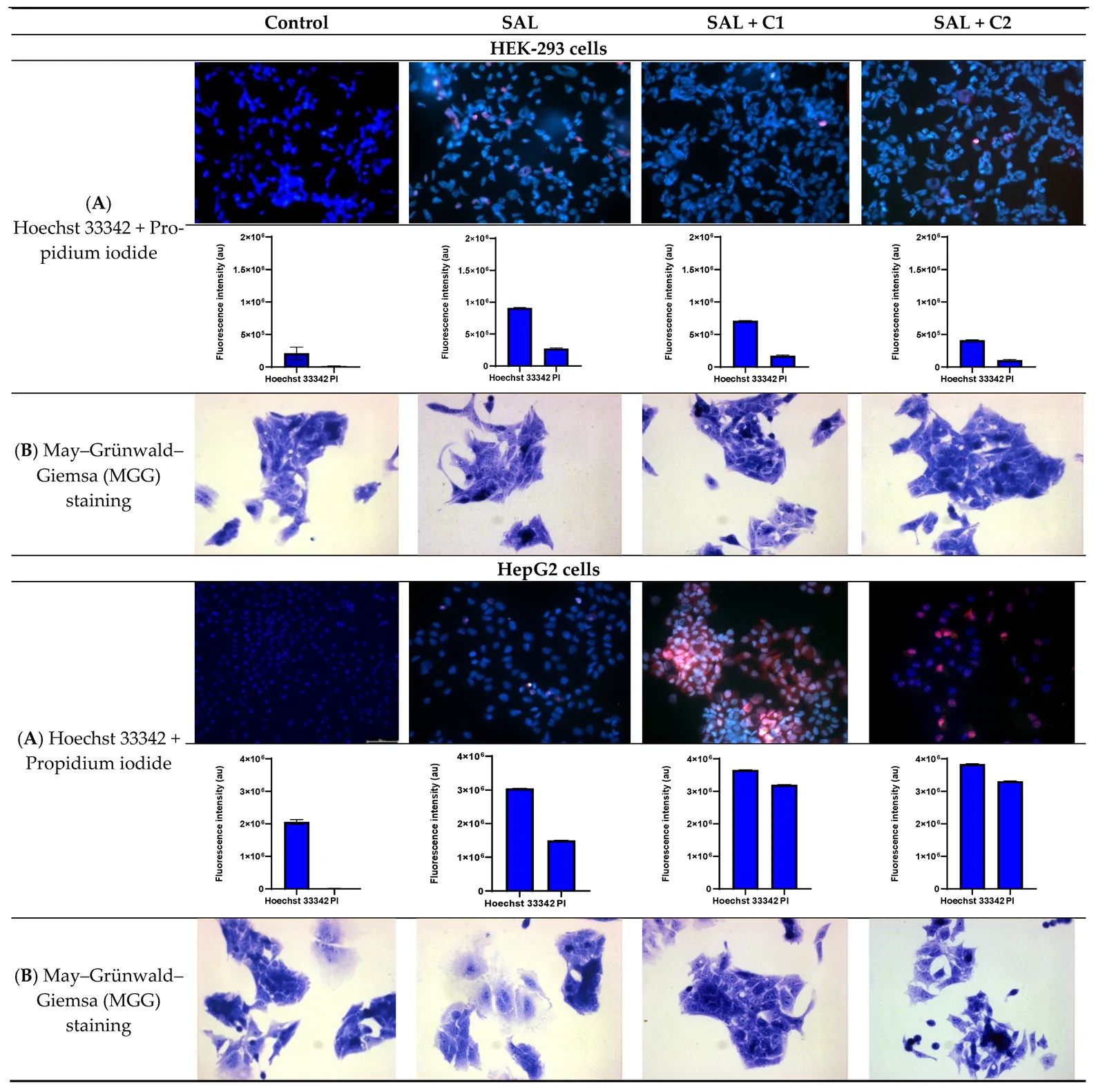

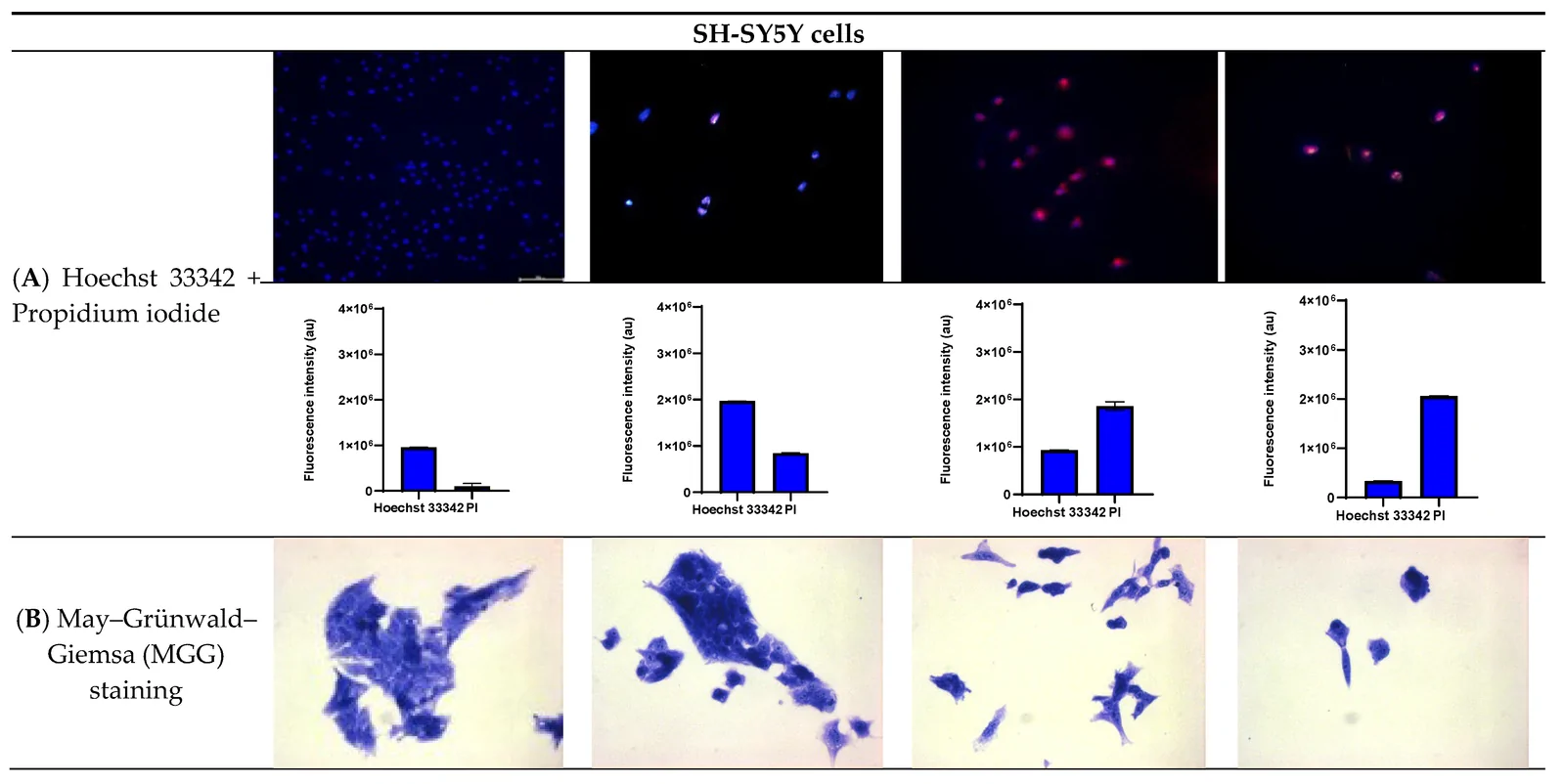
(**A**) Fluorescence images of Hoechst 33342 and propidium iodide staining for each treatment group after 72 h of exposure; the accompanying column graphs show the median fluorescence intensity of each dye. Scale bars correspond to 100 μm. (**B**) Morphological evaluation of HEK-293, HepG2, and SH-SY5Y cells (control vs. treatments: SAL—salinomycin; SAL + C1—salinomycin with cannabidiol at 1.56 μg/mL; SAL + C2—salinomycin with cannabidiol at 3.125 μg/mL) after 72 h of incubation using May–Grünwald–Giemsa (MGG) staining (200× magnification). Control refers to cells incubated with vehicle (DMSO)-containing medium only, without SAL or CBD (negative control, NC); no positive control (PC) was included in this analysis. All micrographs within a given staining procedure were acquired using identical exposure, gain and illumination settings, and scale bars correspond to 100 μm. Representative images were selected as those in which cell density and morphology corresponded most closely to the mean values obtained for the given treatment in three independent experiments. The quantities plotted are the median fluorescence intensities of Hoechst 33342 and propidium iodide, determined as described in Section 4.9; no counts or percentages of apoptotic and necrotic cells are reported.

In HEK-293 cells, salinomycin significantly increased the median fluorescence intensity of Hoechst 33342 (more than 4-fold) and of propidium iodide (20-fold) relative to the negative control. Compared with salinomycin alone, the SAL + C1 and SAL + C2 mixtures reduced the Hoechst 33342 median fluorescence intensity by 36% and 61%, respectively. Throughout this section, the quantities reported are the median fluorescence intensities of Hoechst 33342 and propidium iodide measured across the cells analysed in the stained images, as described in Section 4.9; they are referred to as the apoptotic and necrotic median fluorescence intensity and are semi-quantitative indicators of nuclear condensation and of loss of membrane integrity, respectively, rather than counts or percentages of apoptotic and necrotic cells. These results indicate an attenuation of salinomycin cytotoxicity by CBD in this cell line (Figure 5).

Morphological analysis of HEK-293 cultures after exposure to the drug and its mixture with CBD revealed distinct structural changes (Figure 5). Salinomycin inhibited cell growth and caused cells to detach from the culture surface. Additionally, cell rounding and loss of intercellular adhesion were observed. Cells shrank, leaving only thin cytoplasmic bridges between adjacent cells. The presence of CBD in the culture increased cell number and mitigated the changes observed with salinomycin alone.

### 2.3. The Effects of CBD on Salinomycin’s Activity in the HepG2 Cell Line

**Figure 6:**
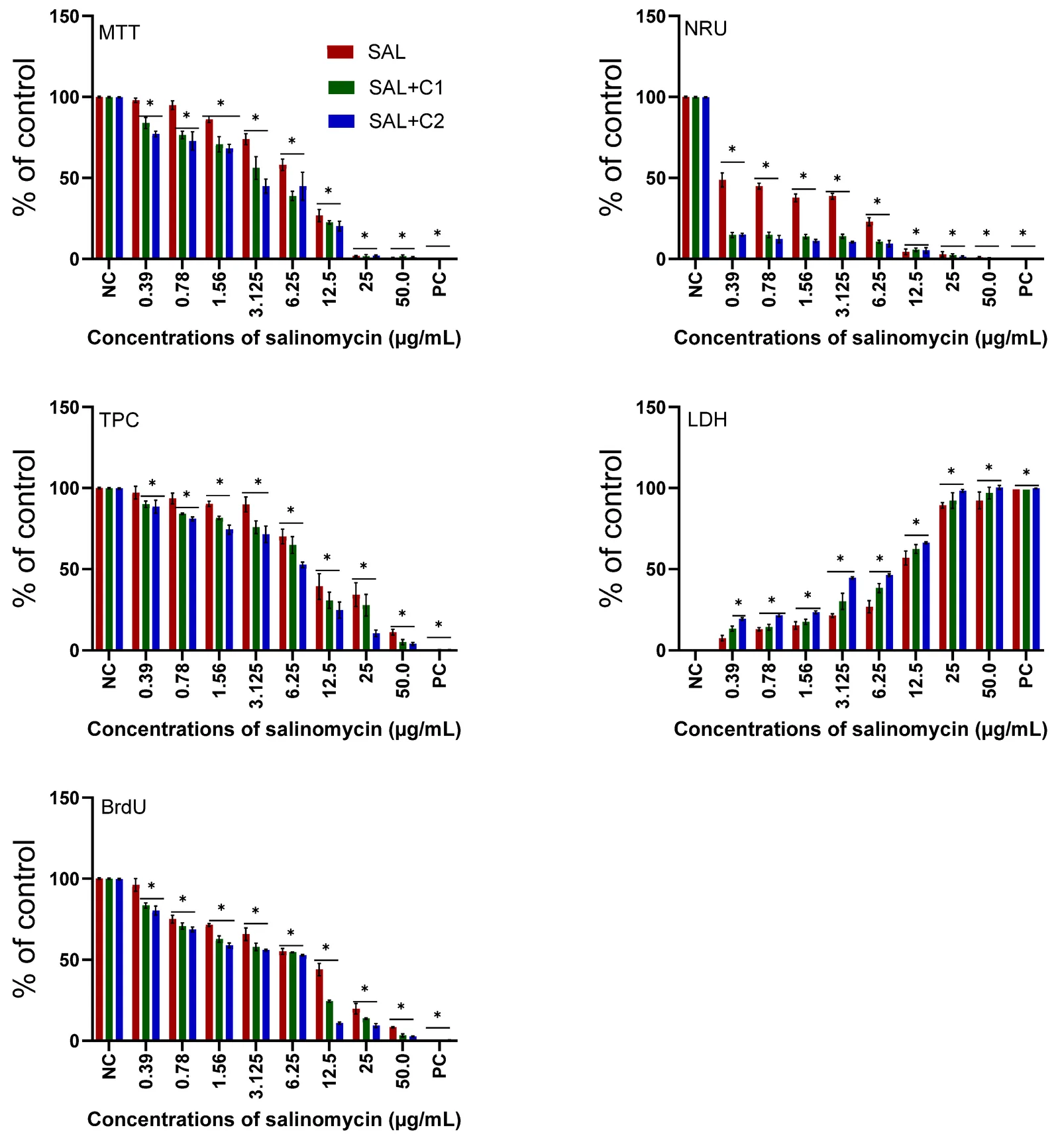
Effects of salinomycin (SAL) alone in the range of 0.39–50 μg/mL (0.50–64.7 μM) and in combination with cannabidiol (CBD) at concentrations of 1.56 μg/mL (4.96 μM; SAL + C1) and 3.125 μg/mL (9.94 μM; SAL + C2) on HepG2 cell lines after 72 h of exposure, assessed using five assays: mitochondrial activity (MTT assay), lysosomal activity (NRU assay), proliferation (TPC assay), cell membrane integrity (LDH assay), and DNA synthesis (BrdU assay). Results are expressed as a percentage of the negative control (NC = 100%) and presented as mean ± SD (standard deviation), *n* = 3 (the number of independent experiments, each performed in six technical replicates per concentration), NC—negative control; PC—positive control. Statistical significance was assessed by comparing each treatment group (SAL, SAL + C1, SAL + C2) with NC. The horizontal line above each concentration group spans the treatment that differed significantly from NC, and the asterisk above the line indicates *p* ≤ 0.05 for that comparison.

Salinomycin inhibited lysosomal activity of HepG2 cells at the lowest tested concentration of 0.39 μg/mL. Higher concentrations of the drug (0.78 μg/mL) inhibited DNA synthesis and caused cell membrane disintegration. A decrease in mitochondrial activity and cell proliferation was observed in HepG2 cells after exposure to 1.56 μg/mL of the drug. The presence of CBD in the mixtures with salinomycin reduced the drug concentration required to elicit effects on all the studied endpoints to 0.39 μg/mL (Figure 6).

Analysis of the IC_50_ values for salinomycin and its two mixtures with CBD (SAL + C1 and SAL + C2) in HepG2 cells revealed that the lowest IC_50_ values were observed in the NRU assay (<0.39 μg/mL) compared to the MTT, TPC, LDH, and BrdU assays. As this value lies below the lowest experimentally tested salinomycin concentration, the IC_50_ for the NRU endpoint in HepG2 cells could not be determined quantitatively; it is reported only as <0.39 μg/mL and was not used for quantitative comparisons. In the MTT assay, a statistically significant decrease in IC_50_ values was observed for both mixtures; in the TPC assay the IC_50_ values of both mixtures were lower than that of salinomycin alone, but the difference did not reach significance. In the LDH and BrdU assays, no significant differences were found between the IC_50_ values of the drug alone and either of its mixtures (SAL + C1 and SAL + C2) (Table 2).

An increase in reactive oxygen species (ROS) production was observed in cultured HepG2 cells starting at the lowest tested concentration of salinomycin (0.39 μg/mL). The presence of CBD in both mixtures induced higher ROS production than the drug alone (Figure 3).

In HepG2 cells the CI values indicated enhancement of salinomycin cytotoxicity (CI < 0.9) irrespective of the drug and CBD concentrations in both mixtures, and this pattern was consistent across all evaluated assays (Figure 4). It should be noted, however, that in the LDH and BrdU assays the IC_50_ values of the mixtures did not differ significantly from those of salinomycin alone (Table 2). In these cases the CI-based classification reflects the position of the complete dose–response curves rather than a significant shift of the IC_50_ point estimate, and should therefore be interpreted with caution.

Salinomycin induced a 48% increase in the apoptotic median fluorescence intensity and a very pronounced (15-fold) increase in the necrotic median fluorescence intensity in HepG2 cells compared to the control group. Compared with salinomycin alone, the SAL + C1 and SAL + C2 mixtures increased the apoptotic median fluorescence intensity by over 20% and induced a marked, approximately two-fold increase in the necrotic median fluorescence intensity. These results suggest a synergistic (potentiating) effect of CBD on SAL cytotoxicity (Figure 5).

Morphological analysis of HepG2 cell cultures after exposure to the drug and its mixture with CBD revealed distinct structural changes (Figure 5). Salinomycin inhibited the growth of HepG2 cells and caused the formation of cell clusters and their detachment from the culture surface. Additionally, cell rounding and loss of intercellular adhesion were observed. The presence of CBD in the culture increased the number of cell clusters and potentiated the observed changes compared to salinomycin alone. In the SAL + C2 mixture, shrunken cells were observed, often remaining as thin cytoplasmic bridges (Figure 5).

### 2.4. The Effects of CBD on Salinomycin’s Activity in the SH-SY5Y Cell Line

**Figure 7:**
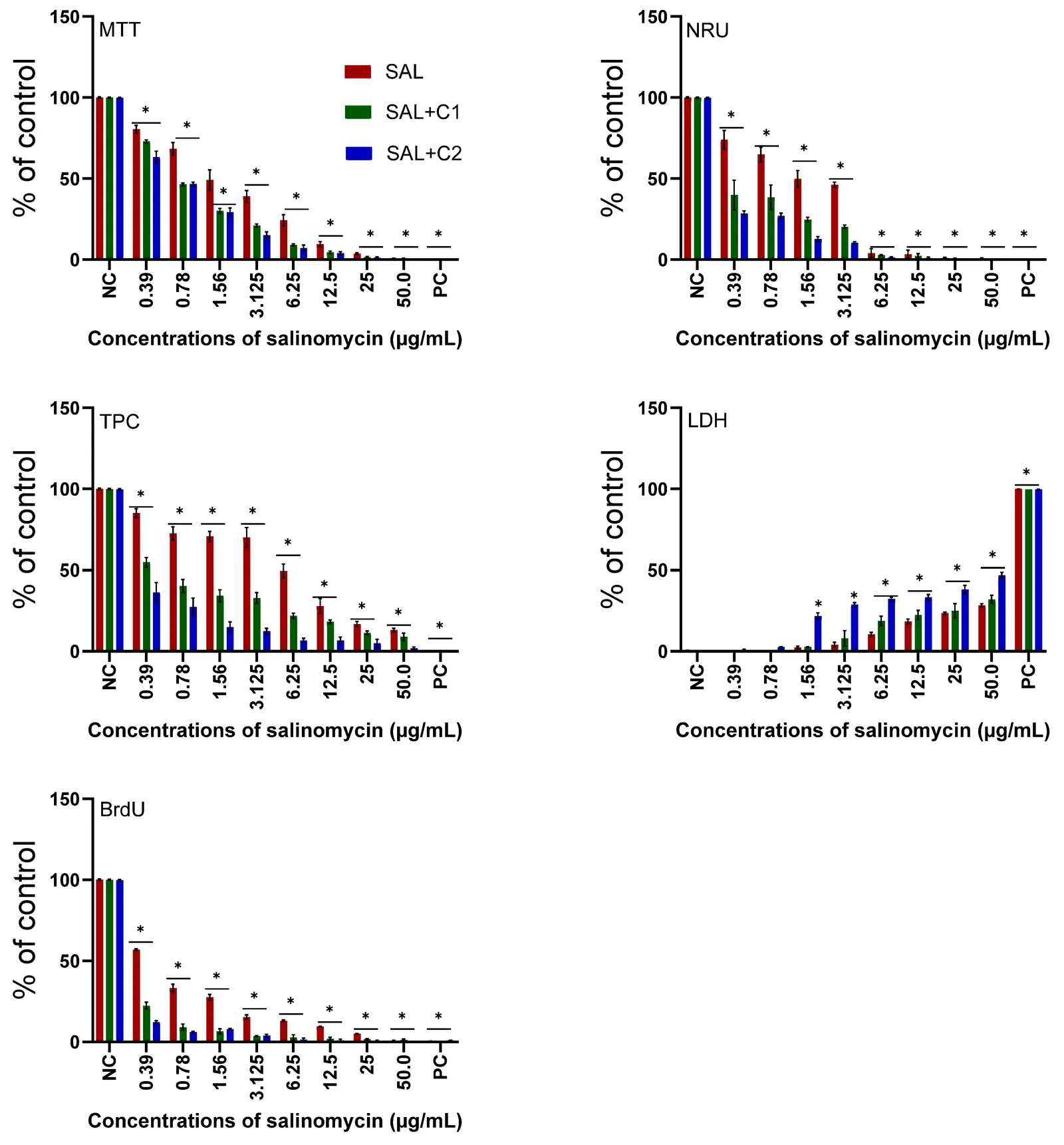
Effects of salinomycin (SAL) alone in the range of 0.39–50 μg/mL (0.50–64.7 μM) and in combination with cannabidiol (CBD) at concentrations of 1.56 μg/mL (4.96 μM; SAL + C1) and 3.125 μg/mL (9.94 μM; SAL + C2) on SH-SY5Y cell lines after 72 h of exposure, assessed using five assays: mitochondrial activity (MTT assay), lysosomal activity (NRU assay), proliferation (TPC assay), cell membrane integrity (LDH assay), and DNA synthesis (BrdU assay). Results are expressed as a percentage of the negative control (NC = 100%) and presented as mean ± SD (standard deviation), *n* = 3 (the number of independent experiments, each performed in six technical replicates per concentration), NC—negative control; PC—positive control. Statistical significance was assessed by comparing each treatment group (SAL, SAL + C1, SAL + C2) with NC. The horizontal line above each concentration group spans the treatment that differed significantly from NC, and the asterisk above the line indicates *p* ≤ 0.05 for that comparison.

SH-SY5Y neuroblastoma cells exhibited the highest sensitivity to the tested drug compared to the other cell cultures (HEK-293 and HepG2). Salinomycin, at the lowest tested concentration of 0.39 μg/mL, inhibited all studied endpoints. The presence of CBD in the mixtures with salinomycin significantly enhanced this inhibitory effect (Figure 7).

Analysis of salinomycin IC_50_ values in SH-SY5Y cells revealed lower IC_50_ values in the MTT, NRU, LDH, and BrdU assays than in the TPC assay. A statistically significant decrease in IC_50_ values was demonstrated for the SAL + C1 mixture in the TPC assay, whereas the decrease recorded for both mixtures in the MTT assay did not reach significance; the TPC value for SAL + C2, and the NRU, LDH, and BrdU values for both mixtures, fell below the lowest concentration tested and could not be compared statistically (Table 2). For several endpoints in this cell line, 50% inhibition was exceeded at the lowest concentration tested, so the IC_50_ values lie below the experimental range; these values are reported as <0.39 μg/mL and are not used for quantitative comparison between treatments.

Like the HepG2 cell cultures, an increase in reactive oxygen species (ROS) production was observed in cultured SH-SY5Y neuroblastoma cells, starting at the lowest tested salinomycin concentration (0.39 μg/mL). The presence of CBD in both mixtures led to higher ROS production than the drug alone (Figure 3).

Furthermore, the same interaction type was observed in SH-SY5Y cells as in HepG2 cells; the synergistic nature of the interaction was independent of drug and CBD concentrations in both cell lines. Consequently, strong synergistic effects were observed across all evaluated assays (Figure 4). Salinomycin induced a 2-fold increase in the apoptotic median fluorescence intensity and a very pronounced (8-fold) increase in the necrotic median fluorescence intensity in SH-SY5Y cells compared to the control group. Compared with salinomycin alone, the SAL + C1 and SAL + C2 mixtures reduced the apoptotic median fluorescence intensity by over 50% and 83%, respectively, while inducing a marked, approximately two-fold increase in the necrotic median fluorescence intensity. This combination of a lower apoptotic and a higher necrotic signal indicates a shift towards a more severe, uncontrolled form of cell death.

These results suggest a synergistic (potentiating) effect of CBD on SAL cytotoxicity (Figure 5).

Morphological analysis of SH-SY5Y cell cultures after exposure to the drug and its mixture with CBD revealed distinct structural changes (Figure 5). Salinomycin caused cell rounding, clustering, and loss of intercellular adhesion. The presence of CBD in the culture reduced cell number and exacerbated the observed changes compared to salinomycin alone. In the SAL + C2 mixture, a rapid decrease in cell number was observed, often accompanied by cells with disrupted membranes and diffused cytoplasmic content (Figure 5).

## 3. Discussion

The study is relevant to both veterinary medicine (where salinomycin is widely used as a coccidiostat in poultry production) and human medicine, where salinomycin and cannabidiol are of growing interest as potential anticancer agents. The cell-line-dependent modulation of salinomycin cytotoxicity by CBD observed here (antagonism in HEK-293 cells alongside increased toxicity in HepG2 and SH-SY5Y cells) raises questions about concurrent exposure to the two compounds, both in animals receiving them together through feed and in any prospective therapeutic use of the combination.

The cytotoxicity of salinomycin toward HEK-293, HepG2, and SH-SY5Y cells observed here, manifested as impaired mitochondrial and lysosomal activity and reduced proliferation, is consistent with its established mechanism of action. Salinomycin disrupts the balance of sodium and potassium ions across the plasma and mitochondrial membranes, causing rapid mitochondrial K^+^ efflux, inhibition of oxidative phosphorylation and, consequently, cell death [32]. More recent work links this mechanism to the sequestration of iron within lysosomes, thereby increasing reactive oxygen species (ROS) production via the Fenton reaction and damaging lysosomal membranes [33,34]. The increase in ROS accompanying the decline in viability in our model, most evident at higher salinomycin concentrations, is consistent with oxidative stress contributing to this cytotoxicity, as observed in cells of various origins [35,36]. Causality was not tested here, since ROS was measured but not manipulated. Although this mechanism was originally characterized in the context of the selective elimination of cancer stem cells, mitochondrial and lysosomal dysfunction, increased ROS production, and secondary apoptosis or necrosis have also been implicated in salinomycin toxicity toward non-malignant cells [35], and all three immortalized lines used here proved susceptible.

The literature contains very limited direct data on the cytotoxicity of salinomycin toward the human HEK-293 cell line; however, in vivo studies and case reports are available indicating the kidney as one of the target organs of this compound’s toxicity. The results obtained in the present study, showing a concentration-dependent impairment of HEK-293 cell function, represent some of the first in vitro data directly characterizing the sensitivity of a human kidney-derived cell line to salinomycin. The attenuation of salinomycin-induced cytotoxicity by CBD observed in HEK-293 cells may be mechanistically explained by the antioxidant and mitochondria-stabilizing properties of CBD, which directly oppose the pro-oxidant mode of action of salinomycin. As a polyether ionophore, salinomycin disrupts Na^+^/K^+^ homeostasis across cellular and mitochondrial membranes and sequesters iron within lysosomes, promoting Fenton-reaction-driven ROS generation and consequent lysosomal and mitochondrial membrane damage [32,33]. CBD, by virtue of its phenolic structure, exerts direct free-radical scavenging activity and has been shown to activate PPAR-γ signaling, leading to reduced mitochondrial ROS levels, increased glutathione content, and upregulation of antioxidant metallothioneins in human cells [37]. Additionally, CBD has been reported to enhance mitochondrial metabolic activity through AMPK/PGC-1α/SIRT3 signaling, further supporting organelle resilience under oxidative challenge [38]. Taken together, these complementary mechanisms—iron-dependent ROS generation and ionophore-mediated membrane disruption by salinomycin, counteracted by CBD’s radical-scavenging and mitochondria-protective activity—provide a plausible molecular basis for the reduced apoptotic and necrotic response observed in HEK-293 cells co-exposed to both compounds.

In contrast to HEK-293 cells, the cytotoxicity of salinomycin in HepG2 cells is relatively well described, both in the context of its anticancer potential and of its hepatic metabolism. Salinomycin inhibits proliferation and induces apoptosis in a dose- and time-dependent manner, arresting the cell cycle in G0/G1 and reducing the CD133+ fraction [39], an effect partly attributable to impaired autophagic flux [40]. Most relevant to the present results, Radko et al. showed that HepG2 cells, unlike primary human hepatocytes, do not efficiently metabolize salinomycin and are correspondingly more susceptible to it; in the same study, the CYP450 inhibitor tiamulin synergistically enhanced salinomycin cytotoxicity in all cell types tested, including non-metabolizing fibroblasts, so the pharmacokinetic interaction alone did not account for the effect [41]. Two non-exclusive mechanisms may therefore underlie the synergy observed here: inhibition of CYP450-mediated salinomycin metabolism by CBD, and the intrinsic concentration-dependent activity of CBD in hepatic cells. Distinguishing their relative contributions would require dedicated CYP450 activity assays in this cell line, which were not performed. The second mechanism has direct support in the literature: CBD inhibits mitochondrial respiratory complexes IV and V, reduces mitochondrial membrane potential and induces autophagy in HepG2 cells [42], and triggers caspase-3/GSDME-dependent pyroptosis and apoptosis through activation of the integrated stress response [43]. As salinomycin independently disrupts mitochondrial ion homeostasis and promotes iron-dependent ROS generation [32,33], a convergent mitochondrial and oxidative insult is a plausible basis for the interaction observed in this line.

Interactions between polyether ionophores and antioxidant compounds in this cell line are not uniformly protective. Cybulski et al. reported that silybin reduced the cytotoxicity of monensin, narasin, and salinomycin in HepG2, LMH, and L6 cultures, while combination index analysis showed that the mode of interaction varied with the cell line, silybin concentration, and endpoint assessed [44]. The direction of such interactions is therefore condition-dependent and not predictable from the modulating compound’s antioxidant profile alone, which provides a point of reference for the potentiating effect of CBD observed here.

In HepG2 cells, the IC_50_ values for SAL + C1 and SAL + C2 did not differ significantly from salinomycin alone in either the LDH or the BrdU assay (Table 2), yet the combination index indicated synergy for these endpoints. The two measures answer different questions. The IC_50_ is a single point on the concentration–response curve and is compared between treatments, whereas the combination index compares the effect of the mixture at a given affected fraction with the effect predicted from the separate median-effect parameters of the two agents across the range of fa. A combination can therefore shift the curve too little to significantly alter the IC_50_ while still producing more effect than additivity predicts, particularly where the concentration–response relationship for salinomycin is steep. The combination index values for these endpoints should accordingly be read as indicating a departure from additivity rather than as evidence of a large change in potency.

The cannabidiol concentrations used in the combinations were 3.7- to 65-fold below its median-effect concentrations across the endpoints with a valid median-effect relationship in these cell lines (Table 1), and neither concentration significantly inhibited any viability endpoint when cannabidiol was applied alone (Figure 1). The modulation of salinomycin cytotoxicity reported here is therefore not attributable to an additive contribution of cannabidiol’s own cytotoxicity. Cannabidiol alone, however, significantly increased reactive oxygen species production at both concentrations in all three cell lines by 1.5- to 1.9-fold relative to the negative control (Figure 1), so a contribution to redox balance cannot be excluded. Apoptosis and necrosis were not evaluated for cannabidiol alone at these concentrations, so a contribution at the level of cell-death signaling likewise cannot be excluded. This intrinsic pro-oxidant effect may appear to conflict with the combination data for HEK-293 cells, in which the same cannabidiol concentrations lowered reactive oxygen species production relative to salinomycin alone (Figure 3). The two observations are not mutually exclusive, because they were obtained against very different oxidative backgrounds. In otherwise unchallenged cultures, the modest rise in reactive oxygen species produced by cannabidiol is expressed against a low baseline, so a small absolute increment appears large in relative terms. In cultures exposed to salinomycin, the dominant source of reactive oxygen species is Fenton chemistry driven by the sequestration of iron in lysosomes [33], and against this much larger oxidative load the radical-scavenging and mitochondria-stabilizing activities of cannabidiol [37,38] are the effects that prevail. A compound can therefore raise reactive oxygen species slightly in an unstressed culture while still lowering them appreciably in a culture under ionophore-driven oxidative stress. This reading is consistent with the present data but was not tested directly, since reactive oxygen species were measured and not manipulated in this study.

SH-SY5Y cells were the most sensitive of the three lines to salinomycin. Whereas in HEK-293 and HepG2 cells, the individual endpoints were progressively inhibited with increasing concentration, in SH-SY5Y cells, all measured parameters were already significantly inhibited at the lowest concentration tested (0.39 μg/mL; 0.50 μM). For several endpoints, and for most endpoints in the two mixtures, 50% inhibition was exceeded at that concentration, so the IC_50_ lies below the experimental range and is reported as <0.39 μg/mL (Table 2). SH-SY5Y cells also showed the most pronounced necrotic profile of the three lines, with an 8-fold increase relative to control, and the presence of CBD in the mixtures further enhanced this, in contrast to the antagonism observed in HEK-293 cells. This sensitivity is consistent with reports that salinomycin induces necrosis in neural-origin cells through ROS–p53–cyclophilin-D-dependent signaling [45], and with work in primary dorsal root ganglion neurons and Schwann cells showing that salinomycin, at concentrations effective against cancer stem cells, raises intracellular Na^+^ and secondarily Ca^2+^ through membrane exchangers, activating calpain and caspases and releasing cytochrome c from depolarised mitochondria [46]. The cells used here were undifferentiated and proliferating, so these results establish the high sensitivity of SH-SY5Y neuroblastoma cells rather than that of mature human neurons; the clinical neurotoxicity of salinomycin in cats, horses, and humans [8,10,11] provides additional evidence that nervous tissue is among its target organs. These observations are consistent with neurotoxicity remaining a safety-limiting property of salinomycin in the veterinary context and in any oncological application, and raise the possibility, which would require an in vivo model to test, that concurrent cannabidiol exposure further narrows its already small margin.

CBD acts on several additional targets relevant to redox balance and mitochondrial function, and the present endpoints cannot determine which of these contributes to the attenuation of salinomycin cytotoxicity in HEK-293 cells. Receptor antagonist or knock-down experiments, together with direct analysis of antioxidant response pathways, would be required to resolve this. Any translational reading of these results is constrained by the models used. All three lines are immortalized or tumor-derived, and the antagonism observed in HEK-293 cells cannot be taken as evidence that CBD protects normal renal tissue or spares healthy tissue during ionophore-based anticancer treatment. The data do support the conclusion that the direction of the salinomycin–CBD interaction differs between cell lines, which is reason to characterize the combination in primary renal cells and in vivo before either the protective or the potentiating effect is assumed to apply to an animal or a patient.

## 4. Materials and Methods

### 4.1. Chemicals and Reagents

Salinomycin monosodium salt (CAS 55721-31-8; molecular weight 772.98 g/mol; Cat. No. 46729, Sigma-Aldrich, Poznań, Poland) and cannabidiol (CAS 13956-29-1; molecular weight 314.5 g/mol; Cat. No. 85705, PhytoLab GmbH & Co. KG, Vestenbergsgreuth, Germany) were used as analytical standards. Triton X-100, hydrogen peroxide, trypan blue, dimethyl sulfoxide (DMSO), fetal bovine serum (FBS), neutral red dye (NR), coomassie brilliant blue R-250 dye, 3-(4,5-dimethylthiazol-2-yl)-2,5-diphenyltetrazolium bromide (MTT), Hoechst 33342, propidium iodide (PI), diacetyldichlorofluorescein (DCFH-DA), trypsin- ethylenediaminetetraacetic acid (EDTA) and antibiotic solution (10,000 U/mL of penicillin, 10 mg/mL of streptomycin) were purchased from Sigma-Aldrich Co. (Poznan, Poland). All other chemicals were purchased from commercial suppliers and were of the highest purity available.

### 4.2. Cell Cultures

The human cell lines HEK-293 (immortalized embryonic kidney), HepG2 (hepatoma), and SH-SY5Y (neuroblastoma) were purchased from the American Type Culture Collection (ATCC CRL-1573, ATCC HB-8065, and ATCC CRL-2266, respectively). These cells were cultured in Eagle’s Minimum Essential Medium (EMEM) (ATCC). The media were supplemented with 10% FBS, 1% L-glutamine and 1% antibiotic solution. The cells were maintained in 75 cm^2^ cell culture flasks (Thermo Scientific Nunc, Roskilde, Denmark) in a humidified incubator at 37 °C in an atmosphere of 5% CO_2_. The medium was refreshed every two or three days, and the cells were subcultured by 0.25% trypsin–0.02% EDTA after reaching 70–80% confluence. Single cell suspensions were prepared and adjusted to a density of 1 × 10^5^ cells/mL (HEK-293 and HepG2), and 2 × 10^5^ cells/mL (SH-SY5Y). The cell suspension was transferred to 96-well plates (100 μL/well) or Lab-Tek chamber slide 8 wells (Nunc) (for staining) and incubated for 24 h before the exposure to the studied compounds or their mixtures. SH-SY5Y cells were used in the undifferentiated, proliferative state; no neuronal differentiation protocol was applied.

Stock solutions of salinomycin monosodium salt and cannabidiol were prepared in DMSO. The stocks were then diluted with culture medium to obtain a concentration range of 0.39–50 μg/mL (0.50–64.7 μM) for salinomycin, and 1.56–200 μg/mL (4.96–636 μM) for cannabidiol alone; in the combination experiments, cannabidiol was used at the fixed concentrations of 1.56 and 3.125 μg/mL (4.96 and 9.94 μM). The final concentration of DMSO in the medium was 0.1% for the SAL and the CBD groups and 0.2% for the SAL + C1 and SAL + C2 groups, reflecting the combined contribution of the salinomycin and cannabidiol stock solutions. For the cannabidiol-alone series, the whole concentration range of 1.56–200 μg/mL (4.96–636 μM) was prepared from a single DMSO stock in such a way that the final DMSO concentration was 0.1% (*v*/*v*) at every cannabidiol concentration tested, including the highest; the series was therefore vehicle-matched across its entire range, and its negative control was medium containing the same 0.1% (*v*/*v*) DMSO without cannabidiol. The fractions affected used to derive the D_m_ values in Table 1, and hence the combination index values, were calculated relative to this vehicle-matched control. The medium used for test solutions and control preparation did not contain antibiotics, and the serum concentration was reduced to 1%. As a negative control (NC), cells were incubated with culture medium containing the corresponding vehicle (DMSO) concentration (0.1% or 0.2%, matching the respective treatment group) without salinomycin or cannabidiol; each treatment group (SAL, SAL + C1, SAL + C2) was expressed as a percentage of its own vehicle-matched NC (100%). As a positive control (PC), cells were treated with 1% Triton X-100, which induces complete plasma membrane lysis and represents maximal cytotoxicity (i.e., 0% viability in the MTT, NRU, TPC, and BrdU assays, and maximal LDH release in the LDH assay). Each concentration was tested in six technical replicates within each of three independent experiments; *n* = 3 throughout refers to the number of independent experiments, and statistics were computed on the means of the independent experiments. Cytotoxicity was assessed after 72 h of exposure of the cells to compounds or their mixtures. The medium was not changed during the incubation time.

### 4.3. MTT Assay

The metabolic activity of living cells was assessed by the measurement of the activity of dehydrogenase [47]. After the incubation of the cells with the studied compounds, 10 μL of the MTT solution (5 mg/mL in PBS) was added to each well of 96-well plates and incubated. After 3 h, the MTT solution was removed, and the intracellular formazan crystals were dissolved in 100 μL DMSO. The plate was shaken for 15 min at room temperature and transferred to a microplate reader Synergy 2 Multi-Mode Microplate Reader (BioTek^®^ Instruments Inc., Winooski, VT, USA) to measure the absorbance at 570 nm, using a blank as a reference. Cytotoxicity was expressed as a percentage of the negative control [20].

### 4.4. NRU Assay

This method is based on staining living cells with neutral red, which readily diffuses through the plasma membrane and accumulates in lysosomes [48]. After the incubation, the medium containing the tested substance was removed and the cells were washed with PBS. Then, 100 μL/well of NR solution (50 μg/mL) was added for 3 h. After this time, the cells were washed with PBS. The dye from viable cells was released by extraction with a mixture of acetic acid, ethanol and water (1:50:49, *v*:*v*:*v*). After 10 min of shaking, the absorbance of the dissolved NR was measured using a Synergy 2 Multi-Mode Microplate Reader (BioTek^®^ Instruments Inc., Winooski, VT, USA) at 540 nm using a blank as a reference. Cytotoxicity was expressed as a percentage of the negative control [20].

### 4.5. TPC Assay

This assay is based upon staining the total cellular protein (proliferation) [49]. After the incubation, the medium containing the drug was removed, and 100 μL Coomassie brilliant blue R-250 solution was added to each well. The plate was shaken for 10 min. Then, the stain was removed, and the cells were rinsed twice with 150 μL of washing solution (glacial acetic acid/ethanol/water, 5:10:85, *v*:*v*:*v*). After that, 100 μL of the desorbing solution (1 M potassium acetate) was added, and plates were shaken again for 10 min. The absorbance was measured at 595 nm in a microplate reader Synergy 2 Multi-Mode Microplate Reader (BioTek^®^ Instruments Inc., Winooski, VT, USA) using a blank as a reference. Cytotoxicity was expressed as a percentage of the negative control [20].

### 4.6. LDH Leakage Assay

The integrity of the plasma membrane was assessed through the test of lactate dehydrogenase (LDH) release [50], which was monitored using the commercially available Cytotoxicity Detection Kit (LDH) (Roche Diagnostics, Warsaw, Poland). Cell-free culture supernatant (100 μL/well) was transferred into the corresponding wells of an optically clear 96-well flat-bottom microplate, and 100 μL reaction mixture was added to each well. Then, the plates were incubated for 30 min at room temperature in the dark. After that time, 50 μL/well of 1 M HCl was added to stop the reaction. The absorbance was measured at 492 nm using a Synergy 2 Multi-Mode Microplate Reader (BioTek^®^ Instruments Inc., Winooski, VT, USA), with a blank as the reference [20].

### 4.7. BrdU Assay

The cell proliferation was evaluated by BrdU assay using the Cell Proliferation ELISA, BrdU (colorimetric) (Roche, Basel, Switzerland), according to the manufacturer’s instructions. Briefly, BrdU was incorporated in the place of thymidine into the DNA of cell under division. This BrdU was detected by an antibody anti-BrdU conjugated with peroxidase, the reaction product of the enzyme with the given substrate was quantified by measuring its absorbance. The absorbance was measured at 370 nm and 492 nm in the microplate reader Synergy 2 Multi-Mode Microplate Reader (BioTek^®^ Instruments Inc., Winooski, VT, USA), using a blank as a reference.

### 4.8. DCFH-DA Assay

The intracellular levels of ROS were evaluated using the redox-sensitive fluorescent dye DCFH-DA [51]. In brief, the cells were incubated with 5 μM DCFH-DA for 1 h at 37 °C in the dark. Thereafter, the tested compounds at study concentrations were added. The cells treated with 500 μM hydrogen peroxide (H_2_O_2_) were taken as a positive control. After 72 h of incubation, the fluorescence of 2′,7′-dichlorofluorescein (DCF) was measured. Fluorescence was determined at 485/530 nm using a fluorescence microplate reader (BioTek^®^ Instruments Inc., Winooski, VT, USA). Fluorescence is reported as raw intensity at 485/530 nm and was not normalized to cell number or to viability. At the higher salinomycin concentrations, where viability was substantially reduced, the signal therefore reflects the fluorescence of the cell population remaining in the well and may underestimate ROS production per surviving cell. ROS data at these concentrations are accordingly interpreted qualitatively, as the presence or absence of an increase relative to the negative control, and not as a quantitative measure of per-cell ROS production.

### 4.9. Hoechst 33342/Propidium Iodide Staining and Measurement of Nuclear Fluorescence Intensity

The type and extent of cell death in the control and treated cultures were evaluated by fluorochrome staining with a mixture of Hoechst 33342 and propidium iodide to distinguish apoptotic and necrotic cells [52]. Images were acquired on an Axiovert 200 M inverted microscope stand (Zeiss, Oberkochen, Germany) fitted with an LSM 510 laser-scanning module (Carl Zeiss, Oberkochen, Germany) and were processed using AxioVision v. 4.6 software; the Axiovert 200 M stand was used for acquisition and the LSM 510 module for confocal imaging. In the qualitative description of the stained cultures, nuclei showing pink fluorescence were regarded as necrotic and nuclei showing blue fluorescence (fragmented and/or with condensed chromatin) as apoptotic. This classification served visual assessment only; no counts or percentages of apoptotic and necrotic cells were derived from it, and none of the quantities reported in Section 2 or in Figure 5 depend on it. Images were acquired from four randomly selected fields per well at 100× total magnification, in three independent experiments, with approximately 5 × 10^2^ cells analysed per field. For each treatment, the median fluorescence intensities of Hoechst 33342 and propidium iodide were determined across all cells analysed, and this quantity, rather than a count or percentage of cells, is plotted in Figure 5A and referred to in Section 2 as the apoptotic and necrotic median fluorescence intensity.

### 4.10. Cellular Morphology Analysis, May–Grünwald–Giemsa (MGG) Staining

This method was used to illustrate the changes that occurred in the cell morphology under the influence of the tested compounds. The staining was performed on Lab-Tek plates. The cells at a density of 1 × 10^5^ cells/mL were used. After 72 h of incubation with the examined drugs or without them (control), the culture media was removed and the cells were stained with 1 mL of May–Grünwald dye for 3 min at room temperature. Then, 1 mL of deionized water was added to each plate. After 3 min of incubation at room temperature, the liquid was removed. All the plates were rinsed with deionized water, and then the cells were stained with Giemsa dye (dilution 1:20) for 30 min at room temperature. After this time, the dye was removed and the wells were rinsed with 1 mL of deionized water and then allowed to dry. The images were taken using light microscope (Zeiss, Oberkochem, Germany). All images within each panel of Figure 5 were acquired at identical magnification (200×), exposure time and detector gain, and were processed identically. For each treatment, the micrograph shown is representative of the fields scored in three independent experiments and was selected as the field closest to the mean cell density for that treatment.

### 4.11. Combination Index Analysis Using the Chou–Talalay Method

To determine the type of interaction between salinomycin (SAL) and cannabidiol (CBD), the combination index method of Chou and Talalay [53] was used. The dose–effect relationships of the individual compounds and their combinations were modelled using the median-effect equation, derived from the mass-action law [53]:

fa/fu = (D/D_m_)^m^

where D is the dose of SAL or CBD; fa is the fraction affected by D; fu is the fraction unaffected (fu = 1 − fa); D_m_ is the median-effect dose (equivalent to IC_50_); and m is the coefficient describing the shape of the dose–effect curve (m = 1, m > 1 and m < 1 indicate hyperbolic, sigmoidal and flat sigmoidal curves, respectively).

For the MTT, NRU, TPC and BrdU assays, in which the signal decreases with cytotoxicity, fa was calculated as 1 − (treated/negative control). For the LDH assay, in which the signal increases with cytotoxicity, fa was calculated as (treated − negative control)/(positive control − negative control), with the Triton X-100 control defining maximal LDH release. Dose–response data for CBD alone were obtained over the range 1.56–200 μg/mL in all three cell lines and in all five assays (Figure 1). Dose–response data for SAL alone were obtained over the range 0.39–50 μg/mL (0.50–64.7 μM) in all three cell lines and in all five assays (Figure 2). All dose–effect data were analysed using CalcuSyn, Version 2.0 (Biosoft, Cambridge, UK). For each compound, assay and cell line, the median-effect parameters (D_m_ and m) and the linear correlation coefficient (r) of the median-effect plot were obtained by linear regression of log(fa/fu) against log(D) (Table 1). Only concentrations yielding 0 < fa < 1 were included. Concentrations at which the signal equalled or exceeded the negative control (fa ≤ 0), or at which inhibition was complete (fa ≥ 1), cannot be represented on a median-effect plot and were excluded. The significance of the regression slope and the 95% confidence intervals of D_m_ were determined from the same regression in GraphPad Prism 11.0. The combination design did not have a constant ratio: a concentration series of SAL was combined with each of two fixed CBD concentrations. The data were therefore analysed using the non-constant-ratio option of CalcuSyn, with the dose of each agent entered separately at every combination point. At each combination point, the CI was calculated as:

CI = D_1_/(D_x_)_1_ + D_2_/(D_x_)_2_

where D_1_ and D_2_ are the doses of SAL and CBD in the combination, and (D_x_)_1_ and (D_x_)_2_ are the doses of each agent alone that produce the same fa, calculated from the single-agent median-effect parameters as D_x_ = D_m_[fa/(1 − fa)]^(1/m)^. Salinomycin and cannabidiol were treated as mutually exclusive agents, that is, as acting through a common mode of action for the purposes of the median-effect analysis; the two-term combination index equation given above follows directly from that assumption, and no α·D_1_D_2_/[(D_x_)_1_(D_x_)_2_] term for mutually non-exclusive agents was included. CI values of 0.9–1.1, <0.9 and >1.1 were taken to indicate additive, synergistic and antagonistic effects, respectively [53]. CI values are reported only within the fa range covered by the measured effects for each assay and cell line; no extrapolation beyond this range was performed. For several endpoints in SH-SY5Y cells, the lowest SAL concentration tested already produced more than 50% inhibition, so CI values at low fa are not supported experimentally and are not reported. CI values for every assay, cell line, mixture and SAL concentration are given in Table S1. For three endpoints, the median-effect relationship for CBD was not valid. CI values for these endpoints are reported for completeness but are marked in Table S1 as unreliable: HepG2 MTT and HepG2 NRU: the slope of the median-effect plot did not differ significantly from zero (*p* = 0.053 and *p* = 0.235, respectively); SH-SY5Y TPC: CBD produced less than 20% inhibition at all concentrations tested, giving an extrapolated D_m_ of 2698 μg/mL.

CI values for HEK-293 LDH and SH-SY5Y LDH should also be interpreted with caution. The CBD D_m_ values for these endpoints (144.5 and 203.2 μg/mL, respectively) lie in the upper part of, or beyond, the tested range, and in both cases the 95% confidence interval of D_m_ extends beyond the highest concentration tested.

### 4.12. Statistical Analysis

Cytotoxicity data are presented as mean values ± SD (standard deviation). The assessment of the cytotoxicity data used a one-way analysis of variance (ANOVA) followed by Dunnett’s post hoc test to determine significance relative to the negative control. The concentration necessary for 50% inhibition of cell viability (IC_50_) by the drug or its mixtures with CBD was calculated using GraphPad Prism 11.0 (GraphPad Software, Boston, MA, USA) by fitting a four-parameter logistic function to the normalized concentration–response data; IC_50_ values are presented as mean ± SEM (standard error of the mean). Statistical comparisons among IC_50_ values were performed by analysis of variance (ANOVA) followed by Tukey’s test. Differences were considered statistically significant at *p* ≤ 0.05.

## 5. Conclusions

Salinomycin showed cell-line-dependent cytotoxicity in the three human cell lines examined, with SH-SY5Y cells the most sensitive. Co-exposure to cannabidiol altered cytotoxicity in opposite directions depending on the cell line: antagonism in HEK-293 cells, with higher IC_50_ values, lower ROS production, and reduced median fluorescence intensities for apoptosis and necrosis; and synergy in HepG2 and SH-SY5Y cells, with increased ROS production and more pronounced cell death. The principal finding is therefore that cannabidiol modifies the cytotoxicity of salinomycin in a manner specific to the cell line examined. The mechanisms proposed here (the antioxidant and mitochondria-stabilizing activity of cannabidiol on the one hand, and its concentration-dependent pro-oxidant and pro-apoptotic activity on the other) remain hypotheses consistent with the study findings. The study is limited to three immortalized or tumor-derived cell lines, to selected concentrations and endpoints, and to a single exposure time, and it does not establish what occurs in normal renal, hepatic, or neural tissue. Confirmation in primary cells and in appropriate in vivo models is required before any translational conclusion is drawn. Further studies using complementary experimental models and approaches are needed to confirm and extend the present findings.

## Figures and Tables

**Table 1:** Median-effect parameters for cannabidiol alone at concentrations of 1.56–200 μg/mL (4.96–636 μM); in HEK-293, HepG2, and SH-SY5Y cells. Dm and m were obtained by linear regression of log(fa/fu) against log(D) using all concentrations yielding 0 < fa < 1; *n* is the number of concentrations entering each fit, and r is the linear correlation coefficient of the median-effect plot. P refers to the test of whether the slope of the median-effect plot differs from zero. For the LDH assay, fa was calculated relative to the maximal release defined by the Triton X-100 positive control, so Dm is the concentration that produces 50% of maximal LDH release. Concentrations are given in μg/mL, with the corresponding molar concentrations in square brackets (cannabidiol, M = 314.5 g/mol; 1 μg/mL ≡ 3.179 μM). n.e., not estimable.

Cell Line	Assay	*n*	m	Dm (μg/mL) [μM]	Dm 95% CI (μg/mL)	r	*p*
HEK-293	MTT	8	2.37	14.8 [47.0]	9.7–22.1	0.97	<0.0001
	NRU	8	1.56	17.1 [54.4]	12.9–22.7	0.98	<0.0001
	TPC	8	1.04	35.8 [113.8]	20.4–76.6	0.94	0.0004
	LDH	8	1.04	144.5 ^c^ [459.5]	75.5–421.7	0.96	0.0002
	BrdU	8	2.47	11.6 [36.9]	5.6–21.0	0.94	0.0006
HepG2	MTT	5	3.29	21.9 ^a^ [69.6]	n.e.–61.0	0.87	0.0532
	NRU	5	1.68	6.1 ^a^ [19.4]	n.e.–49.8	0.65	0.2349
	TPC	7	2.37	31.3 [99.5]	16.6–65.9	0.93	0.0021
	LDH	8	1.82	32.8 [104.3]	23.4–48.8	0.98	<0.0001
	BrdU	8	1.90	25.0 [79.5]	14.6–46.7	0.95	0.0003
SH-SY5Y	MTT	8	1.95	12.3 [39.1]	4.3–27.9	0.90	0.0026
	NRU	8	1.57	36.3 [115.4]	31.3–42.7	1.00	<0.0001
	TPC	8	0.50	2697.7 ^b^ [8578]	340–1.0 × 10^7^	0.85	0.0074
	LDH	8	1.39	203.2 ^c^ [646.1]	97.5–736.2	0.95	0.0002
	BrdU	8	1.52	12.9 [41.0]	7.0–22.2	0.95	0.0003

^a^—The slope of the median-effect plot did not differ significantly from zero, so no valid median-effect relationship was obtained. Combination index values for this endpoint are reported in Table S1, but should not be relied upon. ^b^—Dm extrapolated more than thirteen-fold beyond the highest concentration tested; combination index values for this endpoint are reported in Table S1 but should not be relied upon. ^c^—Dm lies in the upper part of, or beyond, the concentration range tested, and its 95% confidence interval extends beyond the highest concentration tested (200 μg/mL); combination index values for this endpoint should be interpreted with caution.

**Table 2:** IC_50_ values (μg/mL) determined in HEK-293, HepG2, and SH-SY5Y cells using the MTT, NRU, TPC, LDH, and BrdU assays after 72 h of exposure to salinomycin (SAL) and its co-incubation with cannabidiol at concentrations of 1.56 μg/mL (4.96 μM; SAL + C1) and 3.125 μg/mL (9.94 μM; SAL + C2). Data are presented as mean ± SEM (*n* = 3 refers to independent experiments). Values are given in μg/mL, with the corresponding molar concentrations in square brackets (salinomycin monosodium salt, M = 772.98 g/mol; 1 μg/mL ≡ 1.294 μM).

Cell Lines	Assay	SAL	SAL + C1	SAL + C2
HEK 293	MTT	4.4 ± 1.1 ^a^ [5.7]	11.3 ± 2.5 ^a^ [14.6]	11.6 ± 3.2 ^a^ [15.0]
	NRU	1.4 ± 0.9 ^a^ [1.8]	1.6 ± 0.5 ^a^ [2.1]	1.6 ± 0.1 ^a^ [2.1]
	TPC	4.8 ± 1.0 ^a^ [6.2]	10.9 ± 2.2 ^a^ [14.1]	11.9 ± 1.9 ^a^ [15.4]
	LDH	3.7 ± 1.2 ^a^ [4.8]	3.2 ± 0.9 ^a^ [4.1]	3.4 ± 0.8 ^a^ [4.4]
	BrdU	4.5 ± 1.0 ^a^ [5.8]	11.1 ± 1.2 ^b^ [14.4]	11.6 ± 2.1 ^b^ [15.0]
HepG2	MTT	10.9 ± 1.3 ^a^ [14.1]	5.1 ± 0.8 ^b^ [6.6]	1.9 ± 0.3 ^b^ [2.5]
	NRU	<0.39 [<0.50]	<0.39 [<0.50]	<0.39 [<0.50]
	TPC	11.9 ± 1.4 ^a^ [15.4]	9.8 ± 2.5 ^a^ [12.7]	8.4 ± 1.3 ^a^ [10.9]
	LDH	10.4 ± 1.7 ^a^ [13.5]	10.2 ± 0.7 ^a^ [13.2]	9.6 ± 0.1 ^a^ [12.4]
	BrdU	12.1 ± 1.7 ^a^ [15.7]	11.6 ± 0.7 ^a^ [15.0]	9.6 ± 0.3 ^a^ [12.4]
SH-SY5Y	MTT	0.8 ± 0.2 ^a^ [1.0]	0.5 ± 0.2 ^a^ [0.6]	0.4 ± 0.1 ^a^ [0.5]
	NRU	0.7 ± 0.2 [0.9]	<0.39 [<0.50]	<0.39 [<0.50]
	TPC	3.2 ± 0.3 ^a^ [4.1]	0.7 ± 0.2 ^b^ [0.9]	<0.39 [<0.50]
	LDH	<0.39 [<0.50]	<0.39 [<0.50]	<0.39 [<0.50]
	BrdU	0.7 ± 0.1 [0.9]	<0.39 [<0.50]	<0.39 [<0.50]

Within each row, values sharing the same superscript letter (^a^ or ^b^) do not differ significantly, whereas values labeled with different letters (^a^ vs. ^b^) differ significantly, comparing the IC_50_ values of SAL, SAL + C1, and SAL + C2 within the same assay and cell line (*p* ≤ 0.05). Significance letters were assigned on the basis of one-way ANOVA followed by Tukey’s test computed on the three independent experiments; the complete ANOVA and Tukey output for every row is provided in Table S2. Values given as <0.39 μg/mL indicate that the IC_50_ was below the lowest tested salinomycin concentration and could not be determined quantitatively; these endpoints were excluded from quantitative comparisons.

## Data Availability

The original contributions presented in this study are included in the article/Supplementary Materials. Further inquiries can be directed to the corresponding authors.

## Supplementary Materials

The following supporting information can be downloaded at: https://www.mdpi.com/article/10.3390/molecules31214039/s1, Table S1: Combination index (CI) values for salinomycin combined with cannabidiol at 1.56 μg/mL (SAL + C1) and 3.125 μg/mL (SAL + C2) in HEK-293, HepG2, and SH-SY5Y cells, determined after 72 h of exposure using the MTT, NRU, TPC, LDH, and BrdU assays. CI < 0.9 indicates synergy, CI = 0.9–1.1 an additive effect, and CI > 1.1 antagonism. Values are shown for each salinomycin concentration tested;—indicates a concentration not evaluated in that cell line; Table S2: One-way ANOVA and Tukey post hoc output for the comparisons of IC_50_ values presented in Table 2. For every cell line and assay, the IC_50_ values obtained for salinomycin alone (SAL) and for its mixtures with cannabidiol at 1.56 μg/mL (SAL + C1) and 3.125 μg/mL (SAL + C2) were compared by one-way analysis of variance followed by Tukey’s honestly significant difference test (*n* = 3 independent experiments per group). Rows of Table 2 in which the IC_50_ of one or more groups fell below the lowest salinomycin concentration tested (reported as <0.39 μg/mL) could not be compared quantitatively and are omitted; for SH-SY5Y TPC only SAL and SAL + C1 entered the analysis. q is the studentized range statistic. Asterisks mark comparisons significant at *p* ≤ 0.05.
