# Supplementary material for: Cannabidiol Differentially Modulates Salinomycin Cytotoxicity in Human Cell Lines
Source: Molecules. 2026 Nov 9;31(21):4039. doi: 10.3390/molecules31214039 (PMC13765209; doi:10.3390/molecules31214039)
Supplement: Supplementary file 1 [file molecules-31-04039-s001.zip › Supplementary Materials Table S1.pdf]

Supplementary Material

Cannabidiol Differentially Modulates Salinomycin Cytotoxicity in Human Cell Lines

Table S1. Combination index (CI) values for salinomycin combined with cannabidiol at 1.56 μg/mL (SAL+C1) and 3.125 μg/mL (SAL+C2) in HEK-293, HepG2, and SH-SY5Y cells, determined after 72 h of exposure using the MTT, NRU, TPC, LDH, and BrdU assays. CI < 0.9 indicates synergy, CI = 0.9–1.1 an additive effect, and CI > 1.1 antagonism. Values are shown for each salinomycin concentration tested; – indicates a concentration not evaluated in that cell line.

**Cell line / assay**	**Mixture**	**0.39**	**0.78**	**1.56**	**3.125**	**6.25**	**12.5**	**25**	**50**
		μg/mL	μg/mL	μg/mL	μg/mL	μg/mL	μg/mL	μg/mL	μg/mL
**HEK-293**									
MTT	SAL+C1	9.51	9.88	9.00	7.95	5.22	3.08	2.27	0.20
MTT	SAL+C2	9.39	9.25	9.15	4.03	3.03	1.43	1.67	0.19
NRU	SAL+C1	6.03	4.62	2.25	2.06	2.05	1.82	1.55	0.17
NRU	SAL+C2	4.59	4.17	2.38	1.96	1.41	1.60	1.66	0.16
TPC	SAL+C1	6.83	6.21	5.83	5.70	4.88	4.24	1.89	1.24
TPC	SAL+C2	6.40	5.85	5.36	5.44	4.30	1.55	1.39	1.09
LDH [b]	SAL+C1	4.02	2.81	3.39	4.81	3.77	2.93	1.58	1.31
LDH [b]	SAL+C2	3.97	2.76	1.74	1.77	1.75	1.89	1.07	0.18
BrdU	SAL+C1	6.13	5.92	5.72	4.74	3.25	2.01	1.14	0.13
BrdU	SAL+C2	5.64	5.13	4.70	3.50	2.60	1.41	1.13	0.15
**HepG2**									
MTT [a]	SAL+C1	–	0.18	0.25	0.18	0.19	0.04	0.01	0.01

MTT [a]	SAL+C2	–	0.22	0.28	0.29	0.13	0.06	0.01	0.01
NRU [a]	SAL+C1	–	0.30	0.24	0.25	0.12	0.50	0.01	0.01
NRU [a]	SAL+C2	–	0.33	0.27	0.28	0.14	0.56	0.02	0.01
TPC	SAL+C1	–	0.01	0.01	<0.01	0.05	0.09	0.06	0.06
TPC	SAL+C2	–	0.15	0.18	0.19	0.17	0.15	0.24	0.07
LDH	SAL+C1	–	0.40	0.70	0.10	0.20	0.20	0.30	0.50
LDH	SAL+C2	–	0.10	0.10	0.20	0.10	0.10	0.10	0.10
BrdU	SAL+C1	–	0.01	0.10	0.10	0.01	0.20	0.10	0.10
BrdU	SAL+C2	–	0.10	0.10	0.10	0.01	0.30	0.10	0.10
**SH-SY5Y**									
MTT	SAL+C1	0.17	0.22	0.19	0.18	0.15	0.05	0.02	0.01
MTT	SAL+C2	0.26	0.22	0.20	0.24	0.17	0.06	0.03	0.01
NRU	SAL+C1	0.34	0.26	0.35	0.26	0.01	0.50	0.01	0.01
NRU	SAL+C2	0.45	0.38	0.47	0.36	0.03	0.56	0.01	0.01
TPC [a]	SAL+C1	0.30	0.32	0.36	0.37	0.15	0.10	0.05	0.04
TPC [a]	SAL+C2	0.49	0.45	0.56	0.58	0.30	0.21	0.12	0.11
LDH [b]	SAL+C1	0.60	0.40	0.70	0.10	0.20	0.20	0.30	0.50
LDH [b]	SAL+C2	0.10	0.10	0.10	0.20	0.10	0.10	0.10	0.10
BrdU	SAL+C1	0.25	0.14	0.01	0.02	0.01	0.01	0.01	0.01
BrdU	SAL+C2	0.35	0.17	0.02	0.02	0.02	0.01	0.01	0.01

[a] Cannabidiol did not yield a valid median-effect relationship for this endpoint (HepG2 MTT and NRU: slope of the median-effect plot not significantly different from zero, P = 0.053 and P = 0.235; SH-SY5Y TPC: less than 20% inhibition at every concentration tested, extrapolated Dm = 2698 μg/mL). The CI values for these endpoints are reported for completeness but should not be relied upon.

[b] The cannabidiol Dm for this endpoint (144.5 and 203.2 μg/mL respectively) lies in the upper part of, or beyond, the concentration range tested, and its 95% confidence interval extends beyond the highest concentration tested; these CI values should be interpreted with caution.
