# Supplementary material for: Cannabidiol Differentially Modulates Salinomycin Cytotoxicity in Human Cell Lines
Source: Molecules. 2026 Nov 9;31(21):4039. doi: 10.3390/molecules31214039 (PMC13765209; doi:10.3390/molecules31214039)
Supplement: Supplementary file 1 [file molecules-31-04039-s001.zip › Supplementary Materials Table_S2.pdf]

Supplementary Material

Cannabidiol Differentially Modulates Salinomycin Cytotoxicity in Human Cell Lines

Table S2. One-way ANOVA and Tukey post hoc output for the comparisons of IC_{50} values presented in Table 2. For every cell line and assay, the IC_{50} values obtained for salinomycin alone (SAL) and for its mixtures with cannabidiol at 1.56 μg/mL (SAL+C1) and 3.125 μg/mL (SAL+C2) were compared by one-way analysis of variance followed by Tukey's honestly significant difference test (n = 3 independent experiments per group). Rows of Table 2 in which the IC_{50} of one or more groups fell below the lowest salinomycin concentration tested (reported as <0.39 μg/mL) could not be compared quantitatively and are omitted; for SH-SY5Y TPC only SAL and SAL+C1 entered the analysis. q is the studentized range statistic. Asterisks mark comparisons significant at $p \leq 0.05$.

Cell line	Assay	ANOVA F (df)	ANOVA p	Tukey comparison	Mean difference (μg/mL)	q	Tukey p
HEK-293	MTT	2.81 (2, 6)	0.1375	SAL vs SAL+C1	-6.90	2.84	0.1909
				SAL vs SAL+C2	-7.20	2.96	0.1709
				SAL+C1 vs SAL+C2	-0.30	0.12	0.9958
HEK-293	NRU	0.04 (2, 6)	0.9635	SAL vs SAL+C1	-0.20	0.33	0.9697
				SAL vs SAL+C2	-0.20	0.33	0.9697
				SAL+C1 vs SAL+C2	0.00	0.00	1.0000
HEK-293	TPC	4.69 (2, 6)	0.0594	SAL vs SAL+C1	-6.10	3.44	0.1116
				SAL vs SAL+C2	-7.10	4.00	0.0673
				SAL+C1 vs SAL+C2	-1.00	0.56	0.9174
HEK-293	LDH	0.07 (2, 6)	0.9370	SAL vs SAL+C1	0.50	0.51	0.9318
				SAL vs SAL+C2	0.30	0.31	0.9747
				SAL+C1 vs SAL+C2	-0.20	0.20	0.9886
HEK-293	BrdU	6.88 (2, 6)	0.0280	SAL vs SAL+C1	-6.60	4.37	0.0488 *
				SAL vs SAL+C2	-7.10	4.70	0.0367 *
				SAL+C1 vs SAL+C2	-0.50	0.33	0.9704
HepG2	MTT	25.80 (2, 6)	0.0011	SAL vs SAL+C1	5.80	6.46	0.0091 *
				SAL vs SAL+C2	9.00	10.02	0.0010 *
				SAL+C1 vs SAL+C2	3.20	3.56	0.0996
HepG2	TPC	0.94 (2, 6)	0.4413	SAL vs SAL+C1	2.10	1.16	0.7069
				SAL vs SAL+C2	3.50	1.93	0.4158
				SAL+C1 vs SAL+C2	1.40	0.77	0.8527
HepG2	LDH	0.15 (2, 6)	0.8611	SAL vs SAL+C1	0.20	0.19	0.9903
				SAL vs SAL+C2	0.80	0.75	0.8589
				SAL+C1 vs SAL+C2	0.60	0.56	0.9171
HepG2	BrdU	1.51 (2, 6)	0.2937	SAL vs SAL+C1	0.50	0.46	0.9427
				SAL vs SAL+C2	2.50	2.32	0.2998
				SAL+C1 vs SAL+C2	2.00	1.86	0.4381
SH-SY5Y	MTT	1.44 (2, 6)	0.3075	SAL vs SAL+C1	0.30	1.73	0.4827
				SAL vs SAL+C2	0.40	2.31	0.3037
				SAL+C1 vs SAL+C2	0.10	0.58	0.9135
SH-SY5Y	TPC	48.08 (1, 4)	0.0023	SAL vs SAL+C1	2.50	9.81	0.0023 *

Significance letters in Table 2 were assigned from these comparisons: within each row of Table 2, groups that do not differ significantly share a letter.
